# Structural Control Over Bicontinuous Emulsions from Solvent‐Transfer Induced Phase Separation Through Nanoparticle Loading

**DOI:** 10.1002/advs.76965

**Published:** 2026-08-13

**Authors:** Jesse M. Steenhoff, Martin F. Haase

**Affiliations:** ^1^ Van 't Hoff Laboratory for Physical and Colloid Chemistry Utrecht University Utrecht The Netherlands

**Keywords:** confocal microscopy, diffusion, emulsion, energy storage, materials science, nanoparticle, nanotechnology, rational design, scaling, strips

## Abstract

Bicontinuous materials comprise an interpenetrating network of two continuous phases, demonstrating great potential for overcoming diffusion limitations in applications such as catalysis and energy storage. Bicontinuous interfacially jammed emulsion gels (bijels) formed via solvent‐transfer induced phase separation (STrIPS) show particular promise because their constituent nanoparticles enable submicron liquid domains. However, the rational design of diffusion pathways in STrIPS bijels remains challenging due to the unknown scaling between the nanoparticle loading and the domain size. Here, this relationship is investigated by varying the nanoparticle content of STrIPS bijels with a more applied geometry than the typical fibres, namely a supported film. Quantitative analysis with confocal microscopy reveals a decrease in the average domain size with the nanoparticle loading, further forming the basis for both an empirical equation that can predict the bijel film structure and an extended theoretical model to interpret observed morphological trends in terms of limited nanoparticle efficiency. By gaining control over the domain size through the nanoparticle loading, these findings advance the use of supported bijel films as functional materials.

## Graphical abstract

The nanoparticle loading enables effective control over the liquid domain size in bicontinuous interfacially jammed emulsion gels (bijels) produced through solvent‐transfer induced phase separation (STrIPS). The relationship between the nanoparticle loading and domain size profiles in STrIPS bijels is resolved through confocal microscopy and explained through an extended theoretical model of particle‐stabilized emulsions.

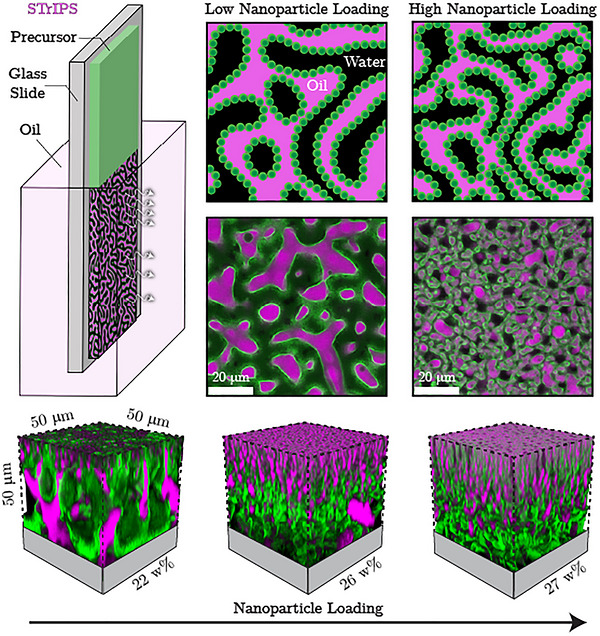

## 1 Introduction

The importance of mass transport through diffusion is difficult to overstate; it drives some of the most relevant and widely applied chemical processes. For example, the supply of reactants to a solid catalyst [1, 2], the movement of lithium ions from the anode to the cathode in a battery [3, 4], or the permeation of gas molecules through a dense polymer membrane [5, 6]. If diffusion takes too long, it can become the rate‐limiting step and lower the efficiency of these processes. Consequently, overcoming the diffusion limitation is crucial for materials applied in catalysis, energy storage, or membrane separation. In this regard, bicontinuous materials hold considerable promise.[Supplementary-material advs76965-supl-0001]

Bicontinuous materials comprise interwoven networks of two distinct phases that together make up the entire volume [7, 8]. Although commonly associated with polymers [9], bicontinuous materials can also be fabricated by arresting the phase separation of immiscible liquids through the interfacial adsorption of nanoparticles [10]. These are known as bicontinuous interfacially jammed emulsion gels (bijels), which offer a host of unique advantages. In particular, their morphology contains a high interfacial area that is readily available for mass transport throughout the entire material [11, 12, 13, 14]. At the same time, the bicontinuous nature effectively prevents unwanted isolation of chemical species through compartmentalization, as can be an issue with high‐surface area emulsions [15]. Finally, the bijel inherently includes nanoparticles that provide further opportunities for functional material design.

The percolating sheet of nanoparticles that delineates its liquid domains is a defining feature of the bijel structure. These nanoparticles can be functionalized for a variety of purposes [16], yet still facilitate the diffusion of chemical species through their interstitial spaces [17]. More important, however, is that the size of the liquid domains depends on the concentration of nanoparticles [10, 18, 19]. In this manner, the nanoparticles thus allow for bijel‐templated materials that overcome the limitations of diffusion by shortening its characteristic path‐length. To achieve this, it is crucial to elucidate the exact relationship between the nanoparticle concentration and the bijel domain size.

For the more common morphology of particle‐laden droplets dispersed in a continuous phase, known as a Pickering emulsion, the inverse relation between the droplet diameter and the particle concentration has been well‐established [20, 21]. This has also been shown for the bijel, where the average size of the liquid domains decreases with increasing particle concentration. In fact, the domain size of the bijel and the inverse volume fraction of the particles obey a distinctly linear relationship [10, 19]. However, this was demonstrated for bijels prepared via temperature‐induced phase separation (TIPS). Although TIPS has the advantage of yielding bijels with uniform domain size distributions, it is also a batch‐based process with limited options for high‐throughput manufacturing. Additionally, the relatively large colloidal particles required for TIPS‐bijels prevent the stabilisation of sufficiently small liquid domains to effectively overcome diffusion limitations [13]. These issues can be addressed by producing bijels through an alternative method, namely solvent‐transfer induced phase separation (STrIPS).

In STrIPS, bijel formation is initiated by the removal of solvent from a precursor mixture, which further consists of immiscible liquids and nanoparticles [22]. This mechanism allows for the continuous fabrication of bijels with volumetric flow rates up to milliliters per minute [23]. Bijels produced in this manner have already found applications as coatings [24], multi‐functional membranes [11, 25] and even scaffolds for tissue engineering [26]. Moreover, extensive research has already been conducted into the morphology of such STrIPS bijels, especially toward nanostructured liquid domains with submicrometer dimensions [13, 14, 27]. This highlighted the influence of the liquid composition and surfactant concentration, yet the role of the nanoparticle concentration remains ambiguous.

Part of this ambiguity is that the liquid domains in STrIPS bijels, in contrast to those produced via TIPS, do not exhibit a single characteristic size. Namely, STrIPS bijels notably feature a distinct gradient in their domain size that originates from the coupled dynamics of solvent‐mediated phase separation and nanoparticle attachment [22, 28, 29]. Previous experimental work already noted the influence of the nanoparticle content on an averaged measure of the domain size [22, 30], yet so‐far only numerical simulations have resolved this effect for the complete domain size gradient present in STrIPS bijels [29].

In particular, the simulations characterized how the nanoparticle content governs the domain size profiles in STrIPS bijel films that are supported by a solid substrate. Compared to fibres typically produced with STrIPS [11, 13, 14], these supported bijel films have the advantage of a mechanically robust substrate, can be produced in larger quantities [23, 31], and have a more common geometry for materials used in energy storage [32, 33] and membrane separation [34, 35]. Consequently, experimental verification of the structural trends observed in the simulations of bijel films is especially important.

As such, the aim of this work is to experimentally establish the relationship between the nanoparticle concentration and the domain size profiles in STrIPS bijel films. To this end, supported bijel films are fabricated via STrIPS with varying nanoparticle content. The resulting morphologies of the bijel films are then reconstructed in 3D through laser‐scanning confocal microscopy. By systematically analyzing the 2D power spectra of confocal cross‐sections, profiles of the liquid domain size are obtained over the depth of the bijel film. These profiles show a marked dependence on the nanoparticle concentration that matches the findings in simulations. Finally, a simple theoretical framework is introduced that explains this dependence for the specific conditions of the STrIPS bijel. Gaining extensive control over the morphology of supported bijel films, specifically the size of the liquid domains, is a crucial step toward the effective implementation of bijel‐templated materials to overcome diffusion limitations in chemical technologies.

## 2 Materials & Methods

### 2.1 Materials

All chemicals were used as received. Diethyl phthalate (DEP, ≥99%), glycerol (≥99%), hydrochloric acid (HCl, 37 wt.%) and toluene (≥99%) were purchased from Thermo Scientific; hexadecyltrimethylammonium bromide (CTAB, ≥99%), 1‐propanol (≥99.5%) and nile red (for microscopy) were acquired from Sigma‐Aldrich; Methanol (HPLC) and n‐hexane (HPLC) were purchased from Biosolve BV; hydrochloric acid (1 M) was obtained from Acros Organics; Silica nanoparticles (Ludox TMA, #1003481587, particle diameter 22 nm) were received from Grace; Microscope slides (cut, uncoated) were purchased from Epredia.

### 2.2 Preparation of Nanoparticle Dispersion & Precursor Mixture

First, a 34 wt.% dispersion of Ludox TMA nanoparticles is concentrated in a rotary evaporator at 60 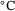 and 140 mbar for 20 min. To remove particle aggregates, the dispersion is then centrifuged (Allegra X‐12R, Beckman Coulter) at 3270× g for 30 min. Afterward, the weight fraction of the nanoparticles in the concentrated dispersion is determined and accordingly diluted to 50 wt.% with ultrapure water (Milli‐Q).

To prepare the precursor mixture, the nanoparticle dispersion is acidified to pH 1.8 with 1 M HCl and immediately added to a combination of DEP, 1‐propanol, glycerol and CTAB. In this precursor, 1‐propanol acts as the solvent, making the oil DEP miscible with the aqueous phase that consists of both the water from the nanoparticle dispersion and glycerol. Following homogenisation, the STrIPS precursor has the following composition: $w_{\mathrm{DEP}}=0.06$, $w_{1\mathrm{prop}}=0.26$, $w_{\mathrm{wat}}=0.29$, $w_{\mathrm{gly}}=0.09$, $w_{\mathrm{NP}}=0.29$, expressed in weight fractions w of the total precursor mixture. An example preparation for 1 mL of precursor is included in the ESI.

The weight fraction of the nanoparticles in the final precursor is controlled by the concentration of the surfactant CTAB. By varying the CTAB concentration, typically between 15 and 40 mM in the entire liquid phase, different degrees of nanoparticle aggregation can be induced in the initial precursor mixture with $w_{\mathrm{NP}}=0.29$. These nanoparticle aggregates are removed by centrifuging the dispersions at 16160× g for 30 min. (Microfuge 16, Beckman Coulter), after which the nanoparticle content of the supernatant is determined by drying at 375 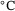 for 24 h. The specific relationship between CTAB concentration and nanoparticle content in the final precursor is discussed in more detail in the Results section.

### 2.3 Fabrication of Supported Bijel Films

Bijel films are formed by coating the precursor on a solid support material, which here are soda lime microscope slides. The microscope slides are first cleaned by immersing them in a 1:1 volume ratio of hydrochloric acid and methanol, which removes any organic contaminants [36]. The cleaned slides are then rinsed with ultrapure water and dried under nitrogen flow.

The supported bijel films are formed via dip‐coating of the cleaned slides. To this end, the slides are sequentially immersed in the precursor mixture and a toluene ambient phase, with the latter step triggering bijel formation. The sample is then prepared for confocal imaging by completely replacing the toluene with dye‐saturated hexane (nile red), which is achieved by immersing the coated glass slides in a volume of hexane significantly exceeding that of the bijel film itself.

## 3 Results

### 3.1 Formation of Supported Bijel Films via STrIPS

**FIGURE 1:**
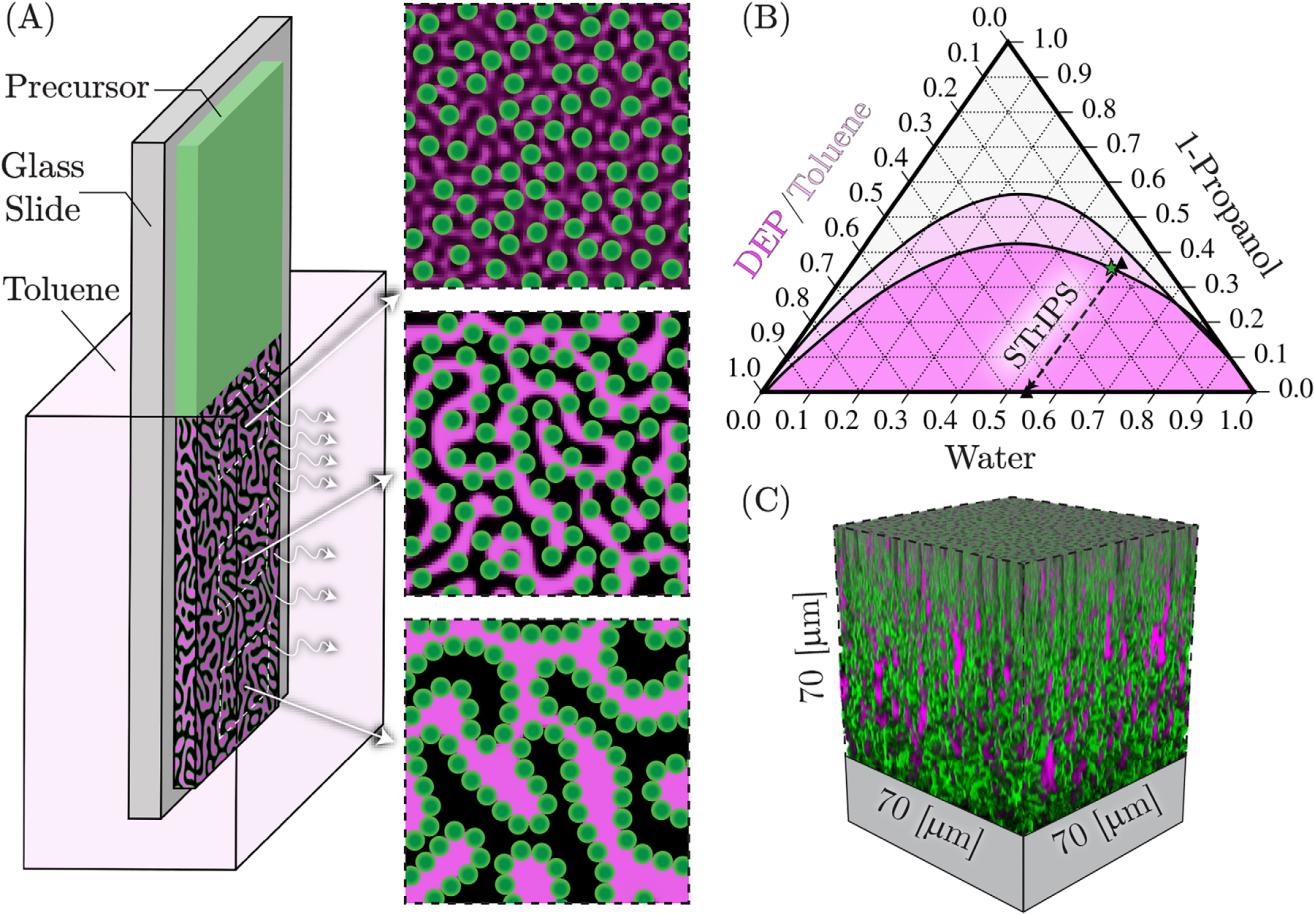
(A) Schematic illustration of the dip‐coating procedure for a supported STrIPS bijel film, highlighting the kinetic arrest of spinodal decomposition through the interfacial attachment of nanoparticles. (B) Ternary phase diagrams of the DEP/water/1‐propanol and toluene/water/1‐propanol [37] liquid mixtures, with the compositions expressed in weight fractions. The solid black lines represent the binodal curves, separating the single‐ and two‐phase regions in the phase diagrams. The two‐phase regions are shaded magenta and light pink for the systems containing DEP and toluene, respectively. (C) Confocal 3D reconstruction of a fabricated STrIPS bijel film, supported by a glass substrate. The magenta color here reflects the presence of oil, whereas water is shown as black, and nanoparticles as green.

A crucial part of this work is the successful implementation of a dip‐coating technique for the facile fabrication of STrIPS bijel films on solid substrates. The experimental procedure, illustrated schematically in Figure 1A, simply amounts to the dip‐coating of cleaned glass slides with the bijel precursor, followed by their immediate immersion in bulk toluene. The solvent subsequently diffuses into the ambient oil, initiating liquid–liquid phase separation in the precursor mixture. The included nanoparticles accumulate on the newly formed liquid interface, eventually forming a dense, jammed layer that effectively arrests further phase separation and stabilizes the emulsion. The final structure of this particle‐stabilized emulsion strongly depends on the initial composition of the precursor mixture and the mass transport during STrIPS, best discussed in the context of the ternary phase diagram shown in Figure 1B.

As the name implies, a ternary phase diagram can be used to characterize the phase behavior of ternary liquid mixtures. In particular, this phase diagram provides information on the systems of DEP/water/1‐propanol, the liquid components in the bijel precursor, in addition to toluene/water/1‐propanol. Here, the latter is included because during STrIPS the DEP in the initial precursor mixture is gradually replaced by toluene from the ambient liquid. Note that neither diagram explicitly considers glycerol, which is also added to the precursor liquids to increase the index‐matching in the final bijel and thereby facilitate confocal analysis [13, 22]. At the concentrations used in this study, glycerol can instead be treated as part of the water fraction without considerable changes in the liquid phase behavior [14, 27].

The binodal curves, shown as solid black lines in Figure 1B, represent the boundaries between the one‐ and two‐phase regions for the DEP/water/1‐propanol and toluene/water/1‐propanol liquid mixtures. The former was experimentally determined by turbidimetry, while the latter was acquired from the literature [37]. The critical point of the mixture containing DEP, where the binodal and spinodal curves are supposed to meet, was also taken from the literature [13, 27] and indicated by the green star. As there is no experimentally determined spinodal curve for the DEP system at present, this critical point is generally identified by tracing the compositions along the binodal curve and noting when the majority and minority phases of the formed emulsion invert.

The formulation of the bijel precursor, listed in the Materials section, is chosen such that the composition of the liquid medium lies close to the critical point. With only the contributions of liquids, ignoring the solid nanoparticles and accounting for the added glycerol through the water fraction, the composition of the liquid medium is given by: $w_{\mathrm{DEP}}=0.08$, $w_{\mathrm{wat}}=0.55$, $w_{1\mathrm{prop}}=0.37$. Indicated by the upper black triangle in the phase diagram, this composition thus represents the bijel precursor prior to STrIPS.

Exposure of the precursor to ambient toluene then induces an extensive transfer of solvent and oils, with both 1‐propanol and DEP diffusing out of the precursor while being replaced by an influx of toluene. By assuming the equivalent exchange between oils and solvent, with details provided in the ESI, the composition of the precursor after the complete depletion of 1‐propanol is given by the lower black triangle in the phase diagram. By connecting the two triangles in the phase diagram, a rough compositional pathway of the STrIPS process can thus be constructed.

The compositional pathway prior to the onset of phase separation is of particular importance for the successful fabrication of STrIPS bijels. Crossing the binodal curve close to the critical point ensures direct entry into the spinodal region of the phase diagram and therefore phase separation through spinodal decomposition, the latter being a key requirement to obtain the characteristic bicontinuous morphology of the bijel. Shifting the point of entry away from the critical point has been linked to the appearance of more “nucleated” structural features due to competing mechanisms of phase separation, such as nucleation and growth [14]. By choosing an initial composition of the bijel precursor very close to the critical point, the compositional trajectory is effectively guaranteed to pass through it as the solvent is removed, compensating for uncertainties in the exact compositional change due to the continuous exchange of DEP with toluene and facilitating the formation of a bicontinuous morphology during STrIPS.

The image in Figure 1C verifies this claim, depicting a 3D confocal reconstruction of a STrIPS bijel film supported by a glass substrate. The oil‐ and water‐rich phases are colored magenta and black, respectively, while the nanoparticles are coloured green. By matching the refractive index of the oil and water phases with glycerol, the morphology of the bijel can be accurately resolved over the entire depth of the film. Consisting of interwoven channels of oil and water, stabilized by nanoparticles, the imaged structure confirms the successful formation of a bijel. Furthermore, the morphology displays one characteristic feature of a STrIPS bijel: the presence of a pronounced gradient in the size of the liquid domains, which grow larger toward the glass substrate. By varying the nanoparticle content in the precursor mixture, both the pore size gradient and the overall size of these liquid domains can be controlled.

### 3.2 Controlling Nanoparticle Content in the Precursor

Following the successful synthesis of a supported bijel film via STrIPS, the nanoparticle loading in the precursor mixture is varied to elucidate its morphological influence. This is done through the concentration of the surfactant CTAB, which governs the stability of the nanoparticles in the relatively apolar liquid medium of the precursor mixture. In particular, the surfactant concentration induces different degrees of nanoparticle aggregation in the initial precursor with $w_{\mathrm{NP}}=0.29$. The aggregates are then removed, yielding stable precursors with different nanoparticle contents.

**FIGURE 2:**
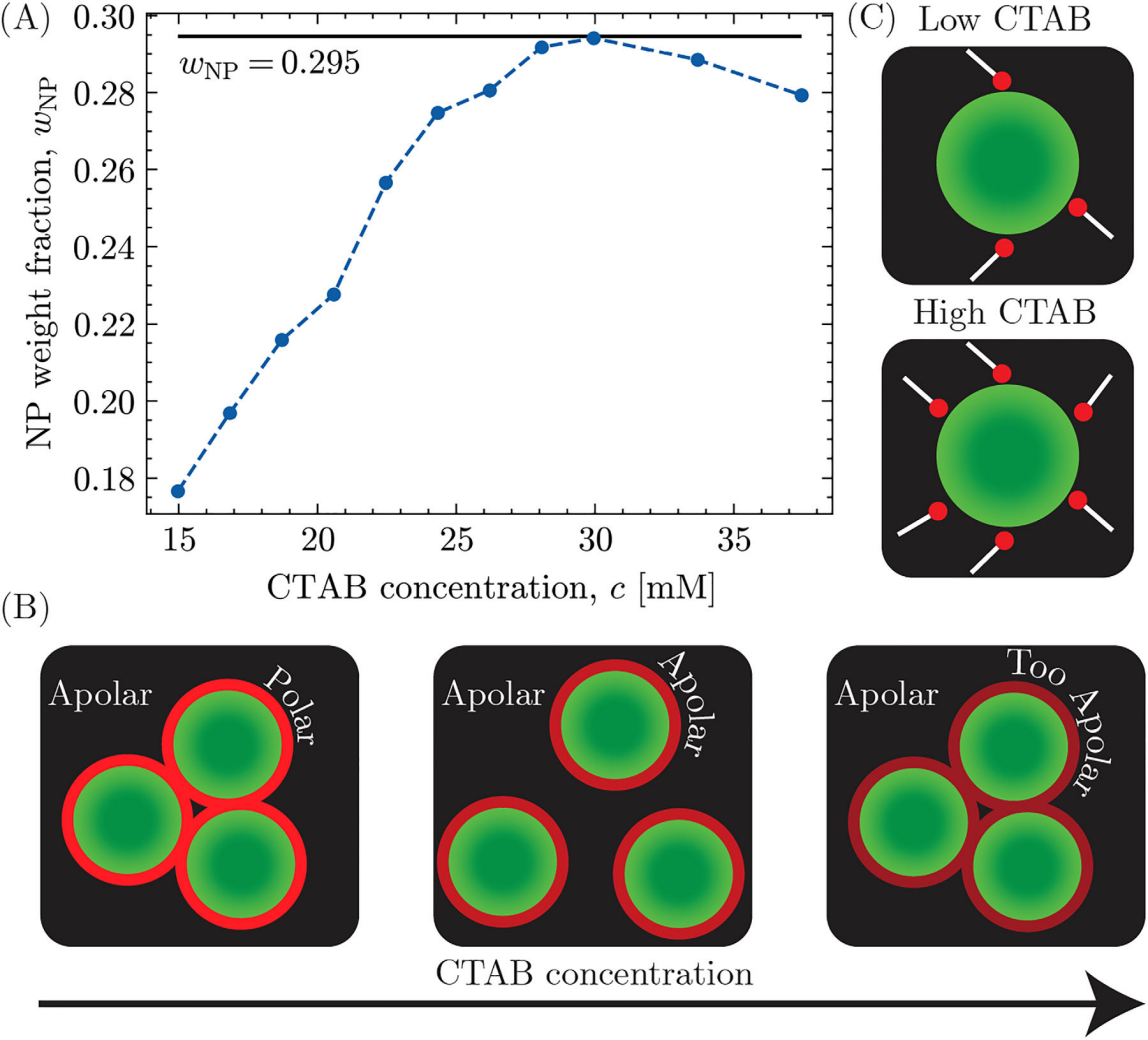
(A) Plot of the nanoparticle weight fraction $w_{\mathrm{NP}}$ against the CTAB concentration c. The solid horizontal line indicates the theoretical maximum, given by the weight fraction in the composition of the initial precursor. (B) Schematic explanation of the observed stability of the nanoparticle dispersion, linking it to differences in the relative polarity of the nanoparticles and the liquid medium of the precursor. (C) Schematic illustration of the hydrophobic functionalization of a silica nanoparticle at different CTAB concentrations. The surfactant assembles with its polar head directed toward the silica surface, leaving its hydrophobic tail sticking out into the liquid.

The specific relationship between the CTAB concentration and the weight fraction of the nanoparticles in the final precursor is illustrated in Figure 2A. Initially, the nanoparticle loading increases with the surfactant concentration, reaching a maximum at 30 mM where nearly all nanoparticles in the initial precursor appear to be stabilized. Any further increase in the CTAB concentration causes the nanoparticle content to decrease again.

The maximum in nanoparticle loading observed in Figure 2A is likely due to the presence of the surfactant CTAB that modifies the relative interactions between individual nanoparticles and the liquid medium of the bijel precursor, a mechanism schematically illustrated in Figure 2B. The surface of bare silica nanoparticles, mostly consisting of disiloxane and silanol groups, is comparatively polar to the liquid medium of the precursor mixture, which contains a significant fraction of apolar oil and solvent. This mismatch in polarity makes bare silica nanoparticles unstable against aggregation in the liquid environment of the precursor.

As visualized in Figure 2C, by introducing CTAB the polar head groups of the surfactant molecules can anchor to the surface of the nanoparticle, imparting a partial apolar character through the hydrophobic tails [13, 27, 38]. With higher CTAB concentrations, the progressively more apolar nanoparticles can initially disperse better in the liquid medium, increasing their weight fraction in the final precursor mixture. However, above the surfactant concentration threshold of approximately 30 mM, the hydrophobic interactions between the surfactant‐functionalized nanoparticles become sufficiently strong to again induce aggregation, which in turn lowers the content of the nanoparticles in the final bijel precursor.

A key feature of this stabilization mechanism is that the surfactant is not necessarily distributed evenly among the nanoparticles. The extent of surfactant functionalization and therefore stability against aggregation depends on the specific surface properties of the nanoparticle. The described method for controlling the loading of the nanoparticles through the concentration of the surfactant effectively isolates the specific fraction of the nanoparticles that are stable under the local conditions of the precursor mixture. More details on how the distribution in surface properties of the nanoparticles influences their interaction with the surfactant CTAB are provided in the ESI.

### 3.3 Morphological Influence of Nanoparticle Content

**FIGURE 3:**
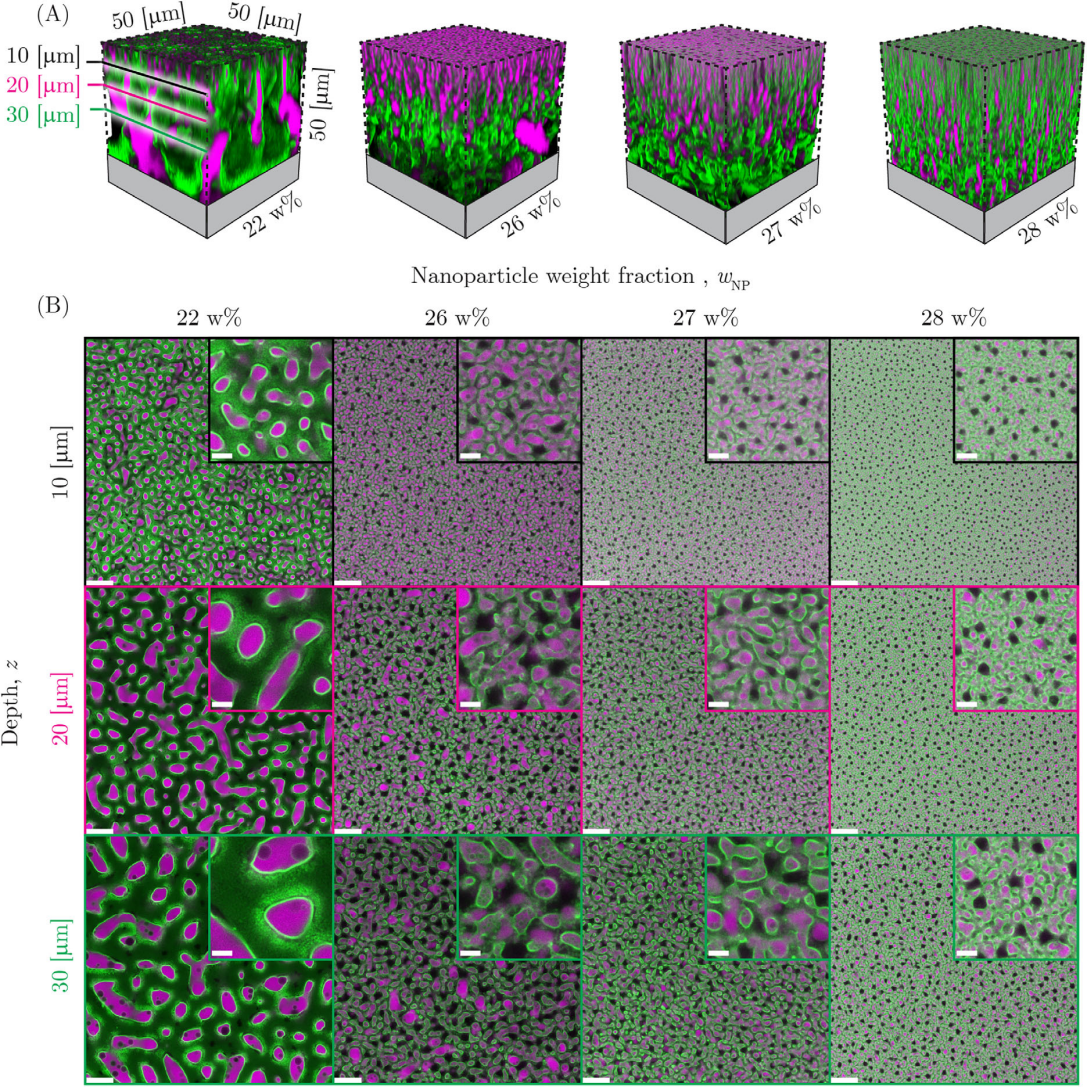
(A) Representative 3D morphologies of STrIPS bijel films, prepared with different nanoparticle weight fractions $w_{\mathrm{NP}}$. For a consistent overview with clear features for all films, only a cubic selection with 50 μm sides is shown. The bijel films are supported on the bottom by a glass substrate, schematically shown in grey. The oil phase is indicated in magenta, the water phase in black and the nanoparticles in green. (B) 2D confocal cross‐sections taken at equivalent depths z between the different STrIPS bijel structures. The insets show representative regions of the image at higher magnification. Each image has dimensions of 184 μm × 184 μm, with a scale bar of 20 μm. For the insets, the dimensions and scale bar are 30.7 μm × 30.7 μm and 5 μm, respectively.

Having established control over the weight fraction of the nanoparticles in the precursor mixture, STrIPS bijel films of varying nanoparticle loading were analyzed through confocal microscopy. The results of this analysis are collected in Figure 3. In Figure 3A, some representative 3D structures of STrIPS bijel films with different nanoparticle weight fractions are depicted. Although all films exhibit a typical STrIPS bijel structure, it is apparent that the nanoparticle content plays a major role in shaping the morphology. Most notably, the average size of the liquid domains visually decreases with increasing nanoparticle loading. Furthermore, the change in domain size over the depth of the structure appears to become more gradual at higher nanoparticle concentrations.

These observations become more evident through the cross‐sections in Figure 3B, taken at equivalent depths among the different STrIPS bijels. For all bijels, the average domain size increases deeper in the structure toward the solid substrate. This gradient, originating from the interplay of solvent‐mediated phase separation and nanoparticle attachment, is an inherent feature of the STrIPS process that is maintained irrespective of the nanoparticle concentration. However, the steepness of the gradient, indicating the rate at which the domain size changes over the depth of the structure, does decrease at higher nanoparticle concentrations. Additionally, comparing the cross‐sections at the same depths shows that the size of the liquid domains considerably decreases at higher nanoparticle weight fractions. With more nanoparticles being able to stabilise smaller domains, this behavior qualitatively matches previous findings for particle‐stabilized emulsions [10, 19, 20, 21].

Apart from these two trends, Figure 3 contains some other morphological features that warrant discussion. The first is the appearance of columnar structures above a nanoparticle weight fraction of 22 wt.%. These pillars, mostly composed of water, extend from the surface of the bijel film deep into the structure. In the 2D cross‐sections of Figure 3B, the water columns are visible as circular voids, notably depleted of nanoparticles. Although their exact origin remains unclear, some preliminary insights can be gained from their general behavior. For example, the water pillars display a specific directionality that differs from the more isotropic bicontinuous structure of the bijel. In particular, the pillars are aligned perpendicularly to the surface of the bijel film, which is along the direction of solvent diffusion during STrIPS. Furthermore, the cross‐sections of the bijel structure indicate that they appear more frequently with increasing nanoparticle content. Together, these observations imply that the formation of the columns is tied with the dynamics of solvent removal during STrIPS, perhaps providing a more direct route to the ambient phase compared to a dense bicontinuous network. However, additional investigation is required to fully elucidate the origin of these directional structures.

The second feature is related to the partitioning of the nanoparticles in the bijel structure. For the lowest weight fraction in Figure 3B, the considerable overlap between the black and green regions in the image suggests that a significant fraction of the nanoparticles occupies the water channels of the bijel, rather than being located at the interface. The overlap seemingly decreases with nanoparticle loading, indicating a redistribution of the nanoparticles toward the interface or even the oil channels of the bijel.

**FIGURE 4:**
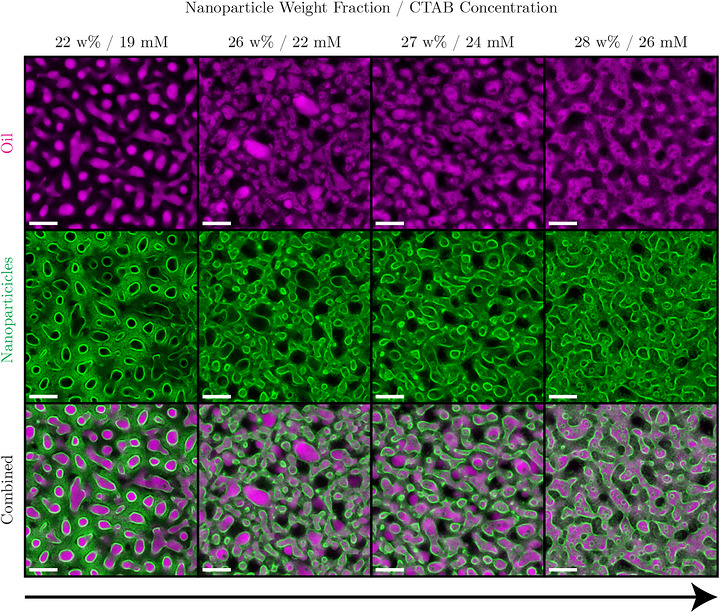
Confocal cross‐sections of STrIPS bijel films with different nanoparticle loadings and the corresponding CTAB concentrations. The fluorescent signals from the oil (magenta) and nanoparticles (green) are shown separately in the top and middle rows, respectively, while the bottom row provides the combined image. The presence of water (black) in the bijel structure is then identified by the absence of signal in the top row of images. To obtain a more uniform size of the liquid domains between different images, cross‐sections were acquired at depths z of 11 μm, 22 μm, 32 μm, 61 μm for the bijel films with nanoparticle weight fractions of 22, 26, 27, 28 wt.%, respectively. The dimensions of each image are 61.3 μm × 61.3 μm, with a scalebar of 10 μm.

To better visualize this transition, the confocal cross‐sections in Figure 4 separately show the fluorescent signal originating from the oil and the nanoparticles, in addition to the combined image. Moreover, by optimizing the depth of the cross‐section in the bijel film, comparable sizes of the liquid domains are achieved between all images despite different nanoparticle loadings. For the lowest weight fraction of 22 wt.%, the images reveal a complete lack of overlap between the fluorescent signals of the nanoparticles and the oil. Consequently, at this weight fraction the nanoparticles are depleted from the oil and mainly reside on the liquid interface and in the water channels. The distinction between oil‐ and water‐rich domains becomes gradually less apparent in the nanoparticle signal at higher weight fractions, confirming the progressive transition of the nanoparticles to the oil channels.

The observed shift in the partitioning of the nanoparticles is likely due to the influence of the surfactant CTAB. Higher weight fractions of nanoparticles, as demonstrated in Figure 2 and by the labels in Figure 4, require higher concentrations of CTAB to remain stable in dispersion. However, these higher concentrations of CTAB also render the nanoparticles increasingly hydrophobic. The nature of the nanoparticles thus changes with their weight fraction, from predominantly hydrophilic at 22 wt.%/19 mM to more amphiphilic at 28 wt.% / 26 mM, prompting their partial relocation to the oil‐rich domains in the bijel.

In summary, the nanoparticle content of STrIPS bijels significantly influences their general morphology. Increasing the nanoparticle weight fraction reduces both the overall size of the liquid domains and their gradient over the structure. Moreover, higher nanoparticle weight fractions shift the distribution of the nanoparticles in the bijel structure from the water‐rich to oil‐rich domains because of the associated increase in the concentration of the surfactant CTAB. Irrespective of these qualitative findings, quantitative analysis is still required to establish a comprehensive relationship between the nanoparticle content and the size of the liquid domains in STrIPS bijels.

### 3.4 Structural Analysis of STrIPS Bijel Films

**FIGURE 5:**
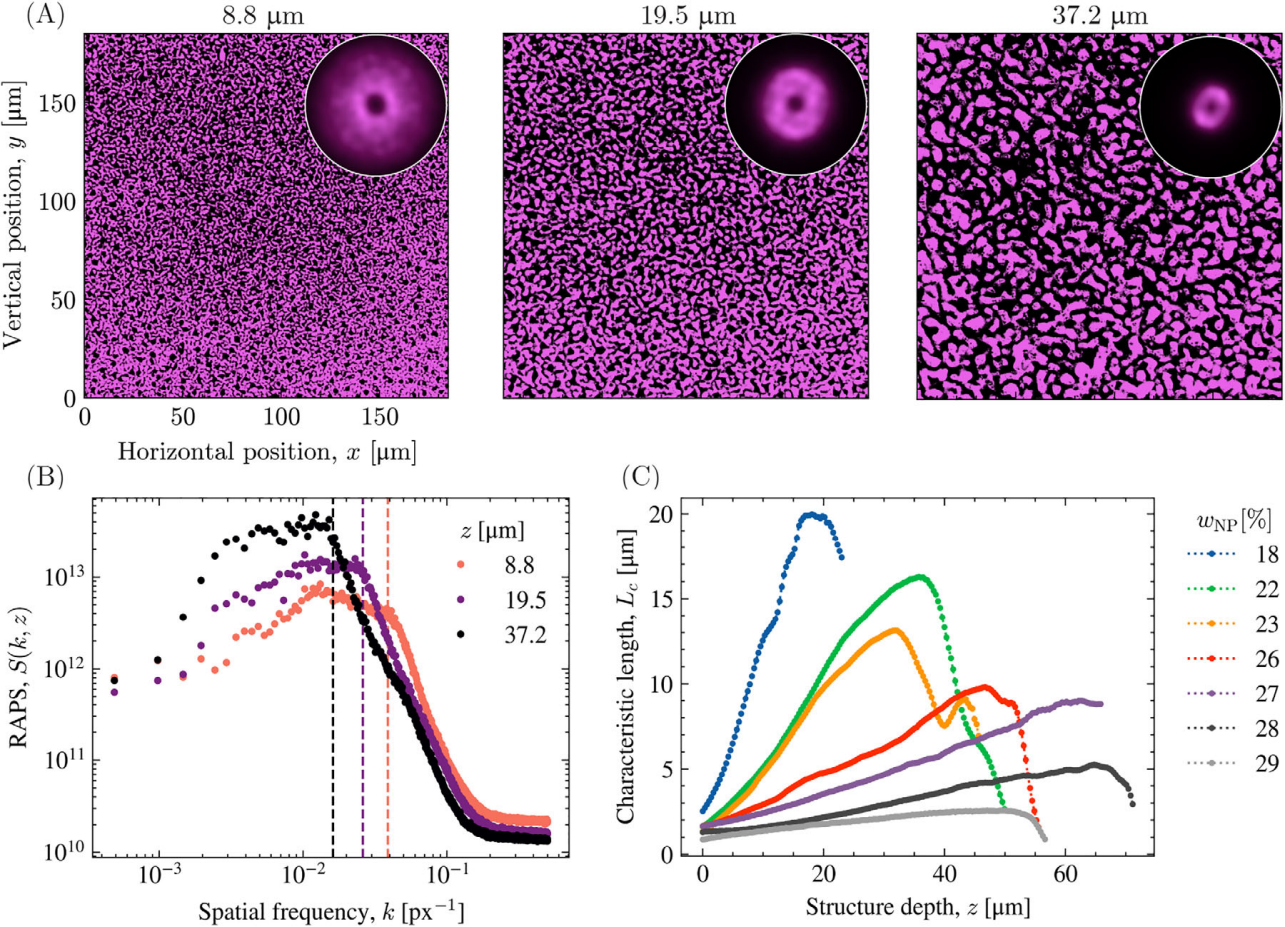
(A) Confocal images taken at different depths in a STrIPS bijel film, containing 27 wt.% nanoparticles. Only the binarized signal from the oil channels is shown here. The inset of each image shows the corresponding 2D power spectrum. (B) Rotationally averaged power spectra (RAPS, S(k,z)) of the images in (A), providing a profile of relevant spatial frequencies at different depths. The vertical lines correspond to the first moments of S(k,z), denoting the characteristic spatial frequency $k_{c}$ at that depth. (C) Profiles of the characteristic length $L_{c}$ over the structure depth z in STrIPS bijel films, with varying nanoparticle weight fractions $w_{\mathrm{NP}}$. Here $L_{c}$ is a measure for the average size of the liquid domains.

To quantitatively analyze the morphological influence of the nanoparticle content, profiles of the liquid domain size across the structure of the STrIPS bijel films are acquired. This is achieved by calculating a characteristic length $L_{c}$ in 2D cross‐sections of the bijel structure, taken at different depths with confocal microscopy. The characteristic length $L_{c}$ is determined from the rotationally averaged power spectrum [39, 40, 41] S(k,z) of these 2D cross‐sections, with the entire process illustrated in Figure 5.

Figure 5A depicts confocal images at different depths z of a STrIPS bijel film, with only the binarized signal from the oil channels shown. Here, the insets contain the associated 2D power spectrum $\hat{\phi }\left(k,z\right)\hat{\phi }\left(-k,z\right)$, calculated from the Fourier transform $\hat{\phi }\left(k,z\right)$ of the oil signal ϕ(x,z). These power spectra reveal the distribution of the spatial frequencies $k=(k_{x},k_{y})$ present in the image, providing valuable information on the local morphology.

For example, the distribution of spatial frequencies in the power spectra is rotationally symmetric and shifts inwards toward the lower frequencies at the centre with increasing depth z. Whereas the latter reflects the well‐known trend that the average size of the liquid domains grows with depth, the former indicates that the structure of STrIPS bijels exhibits in‐plane isotropy. Both support the supposed formation mechanism of STrIPS bijel films: spinodal decomposition of immiscible liquids guided by a planar diffusion front of the solvent [28]. Note that in some cases, as demonstrated by the slightly ellipsoidal shape in the right‐most image of Figure 5A, the power spectra can show minor signs of anisotropy deeper in the bijel film. However, this is likely the result of reduced imaging quality at greater sample depths, rather than the presence of an underlying directional structure.

As the 2D power spectra generally obey circular symmetry, the rotationally averaged power spectrum S(k,z) can be calculated through

(1) $$S\left(k,z\right)={\left\langle \hat{\phi }\left(k,z\right)\hat{\phi }\left(-k,z\right)\right\rangle }_{k},$$

where the outer brackets indicate the rotational average over concentric rings in k‐space at fixed values of k, with k=|k|. As demonstrated in Figure 5B, the rotationally averaged power spectrum S(k,z) provides a convenient profile of the relevant spatial frequencies at all depths in the bijel structure. The dominant spatial frequency $k_{c}$, associated with the characteristic length scale at that depth, is given by the first moment of S(k,z) as

(2) $$k_{c}=\frac{\int kS(k,z)\;dk}{\int S(k,z)\;dk}.$$

The characteristic length $L_{c}$ can then be determined from the inverse of this frequency as

(3) $$L_{c}=\frac{2\pi }{k_{c}}.$$

According to Equation (3) the shift in characteristic frequency $k_{c}$ to lower values with depth z, as visible in Figure 5B, corresponds to an increasing value of characteristic length $L_{c}$. With $L_{c}$ as a measure for the average size of the liquid domains, structural analysis through S(k,z) thus provides quantitative information on the domain size profiles in STrIPS bijels.

The potential of this method is demonstrated in Figure 5C, which contains the depth profiles of $L_{c}$ for STrIPS bijel films with varying nanoparticle content. These profiles reveal that the different bijel films have liquid domains of roughly similar size on their surface. The domains then grow with depth into the bijel structure, eventually reaching a maximum. As qualitatively observed in Figure 3, both the rate of change in the domain size and its maximum value decrease with increasing nanoparticle weight fraction. Interestingly, even deeper in the bijel the average size of the liquid domains decreases again. However, this is caused by the presence of the glass substrate or nucleated sub‐structures that distort S(k,z), as evidenced in the ESI.

In summary, the 3D morphology of STrIPS bijels can be effectively resolved from the power spectra of its constituent 2D cross‐sections, acquired with confocal microscopy. The power spectra offer unique insight into the local morphology, providing information on both size and directionality of the liquid domains. The characteristic spatial frequency is then identified from the rotationally averaged power spectra S(k,z), which enables the construction of profiles for the size of the liquid domains over the depth of the STrIPS bijel film. These profiles corroborate previous qualitative observations, providing a quantitative framework for understanding the role of nanoparticles during STrIPS.

### 3.5 Morphological Scaling of Domain Size and Nanoparticle Content in STrIPS bijels

The results of previous sections demonstrate that the nanoparticles shape the morphological features of STrIPS bijels. This dependence can be quantitatively assessed from the profiles of the liquid domain size over the depth of their structure. The initial region in the profiles is of particular interest, as it reveals the influence of the nanoparticle content on the inward growth of the liquid domains from the bijel surface. For a consistent comparison between bijel films of different thicknesses, this initial region is generally taken as the first 30 μm of the film.

**FIGURE 6:**
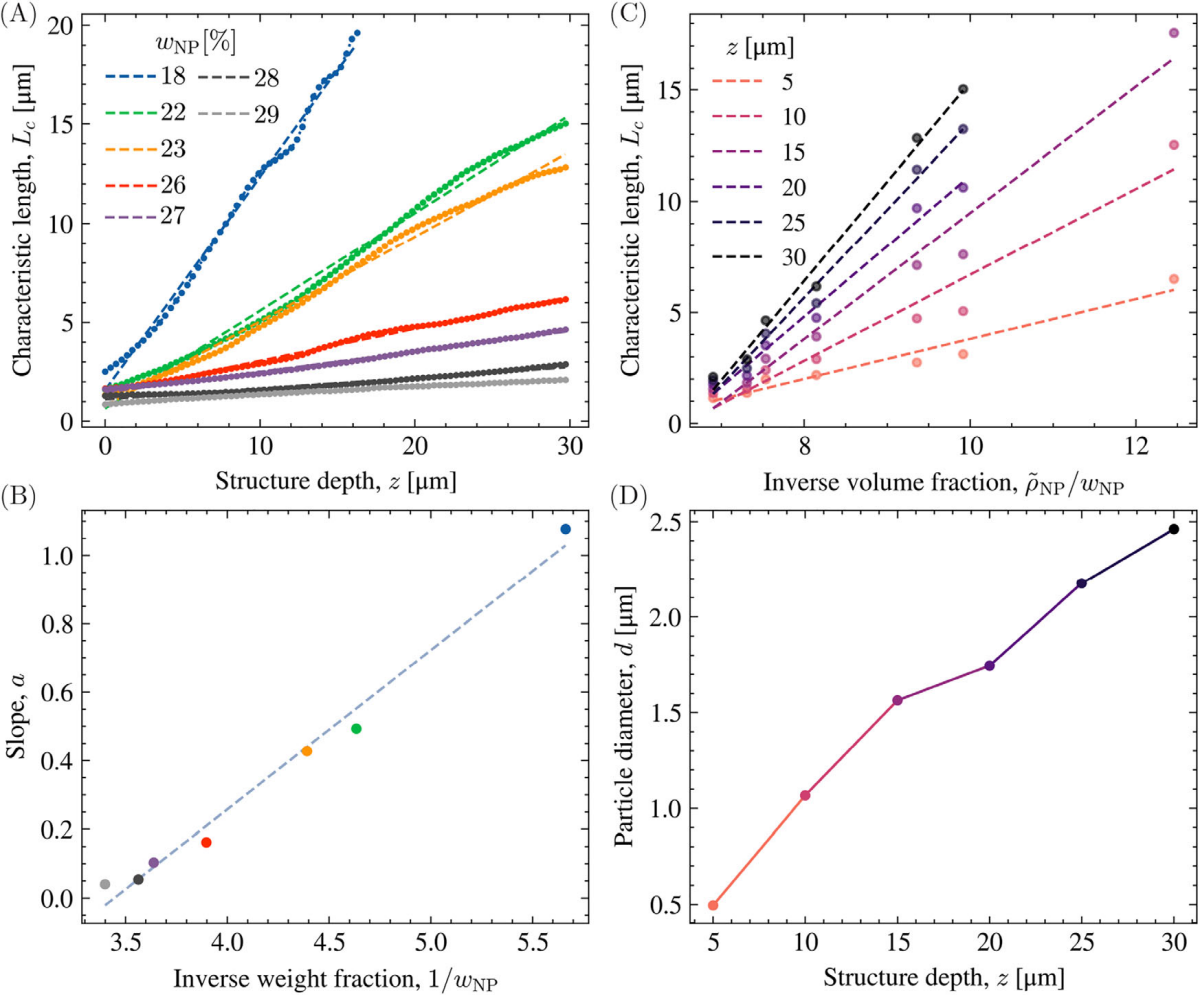
(A) Profiles of the characteristic length $L_{c}$ over the depth z of supported bijel films with varying nanoparticle weight fractions $w_{\mathrm{NP}}$. Here, only the initial region of domain growth from the surface of the bijel is shown. The dashed lines represent linear fits of the profile over this region. (B) Slopes a of the fitted lines in (A) versus the inverse of the nanoparticle weight fraction $1/w_{\mathrm{NP}}$. The dashed line is a linear fit. (C) Plots of the characteristic length $L_{c}$ against the inverse of the nanoparticle volume fraction ${\tilde{\rho }}_{\mathrm{NP}}/w_{\mathrm{NP}}$, sampled at different depths z in the bijel structure. The dashed lines represent fits with Equation (6). (D) The effective particle diameter d at different depths z of the bijel structure. The values of d are calculated from the slopes of the fits in (C) with Equation (6).

Figure 6A isolates these sections of the profiles for the different bijel films. Note that the increase in domain size is distinctly linear and that the average value decreases with the weight fraction of the nanoparticles. These findings broadly agree with those of numerical simulations for STrIPS bijels [28, 29], yet also contain an additional feature that was not readily noticeable before. That is, the slope of the domain size profiles in Figure 6A also strongly depends on the weight fraction of the nanoparticles, with higher nanoparticle loadings resulting in flatter profiles. This relationship is further refined in Figure 6B, showing that the slope increases linearly with the inverse of the weight fraction.

By establishing a connection between the slope of the domain size profiles and the weight fraction of the nanoparticles, an empirical equation can be formulated for the morphology of STrIPS bijel films. Taking into account that the profiles in Figure 6A tend toward a similar size for the liquid domains on the surface of the bijel film, the linear relationships in Figure 6A,B can be combined to yield

(4) $$L_{c}\left(z\right)=L_{c}^{0}+Az+\frac{Bz}{w_{\mathrm{NP}}},$$

where $L_{c}^{0}=1.6\pm 0.5\;\mu m$ is the average surface size of the liquid domains, while A=−1.44 and B=0.42 are empirical fitting parameters. Note that Equation (4) is by no means general or complete. For example, the limited resolution of confocal microscopy leads to an overestimation of $L_{c}^{0}$, with scanning electron microscopy (SEM) revealing a typical size of only a few hundred nanometres for the liquid domains at the surface of STrIPS bijels [13, 14, 42]. In addition, the accurate use of Equation (4) is confined to the range of weight fractions included in this study. However, since this range covers the weight fractions most feasible for the STrIPS process, Equation (4) can still serve as an initial guide to tailor the morphology of STrIPS bijel films.

In addition to characterising the bijel structure, the profiles in Figure 6A contain information on the properties of the nanoparticles. By comparing the dependence of the domain size $L_{c}$ on the nanoparticle loading $w_{\mathrm{NP}}$ with the expected behavior of a model system, a rough approximation of the diameter d of the nanoparticles can be determined. One of the simplest model systems consists of spherical droplets covered by a close‐packed monolayer of nanoparticles. In that case, the relationship between $L_{c}$ and $w_{\mathrm{NP}}$ is given by [10],

(5) $$L_{c}=\frac{\pi d}{\sqrt{3}}\frac{{\tilde{\rho }}_{\mathrm{NP}}}{w_{\mathrm{NP}}},$$

where the more common inverse of the volume fraction $1/\phi_{\mathrm{NP}}$ has instead been expressed as ${\tilde{\rho }}_{\mathrm{NP}}/w_{\mathrm{NP}}$. Here, ${\tilde{\rho }}_{\mathrm{NP}}=\rho_{\mathrm{NP}}/\rho_{sys}$ is the density ratio of the nanoparticles and the entire system, the latter thus including both liquids and nanoparticles. According to Equation (5), plotting $L_{c}$ against ${\tilde{\rho }}_{\mathrm{NP}}/w_{\mathrm{NP}}$ should yield a line with a slope that is set by the diameter d of the nanoparticles. Although Figure 6C shows that $L_{c}$ indeed varies linearly with ${\tilde{\rho }}_{\mathrm{NP}}/w_{\mathrm{NP}}$ for STrIPS bijels, it also raises several points that need to be addressed.

First, none of the lines in Figure 6C pass through the origin, as expected from Equation (5). Instead, the lines indicate the existence of a maximum weight fraction $w_{max}$ that is given by their off‐sets. This implies that above $w_{max}$ the liquid domains reach a size that cannot be resolved by the methods in this work. There, the size of the particle‐stabilized domains likely approaches the minimum dictated by the dimensions of the particles themselves. The value of this maximum weight fraction can be found by introducing an empirical off‐set to Equation (5)

(6) $$L_{c}=\frac{\pi d}{\sqrt{3}}\left(\frac{{\tilde{\rho }}_{\mathrm{NP}}}{w_{\mathrm{NP}}}-\frac{1}{\phi_{max}}\right),$$

which gives a maximum volume fraction $\phi_{max}=0.16\pm 0.01$ when fitted to the data in Figure 6C. In turn, this $\phi_{max}$ can be converted into $w_{max}=0.34\pm 0.02$ for a representative value of ${\tilde{\rho }}_{\mathrm{NP}}=2.2$, suggesting that the size of the liquid domains in STrIPS bijel films can be effectively minimized with nanoparticle weight fractions around 34%.

Second, the characteristic structural gradient in STrIPS bijels means that a particular weight fraction of the nanoparticles does not correspond to a single defining length‐scale. Rather, the relationship between domain size and nanoparticle loading can be sampled at different depths in the bijel film. Although Equation (6) implies equivalent scaling between $L_{c}$ and ${\tilde{\rho }}_{\mathrm{NP}}/w_{\mathrm{NP}}$ for a constant nanoparticle diameter, Figure 6C shows that the slope increases with sampling depth. As such, the effective properties of the nanoparticles vary over the thickness of the bijel film.

Finally, whereas Equation (6) gives a good approximation of the particle diameter d for bijels produced with TIPS [10, 19], it differs by up to two orders of magnitude for the STrIPS bijel. This is illustrated in Figure 6D, showing that the values of d extracted from the slopes of the lines in Figure 6C correspond to micrometer‐sized particles rather than nanoparticles. Moreover, Figure 6D demonstrates that the effective size of the nanoparticles increases with depth, suggesting a lowered efficiency in the stabilization of liquid domains.

These findings demonstrate a strong link between the nanoparticle content and the structure of STrIPS bijels; quantitative analysis even allows the empirical prediction of the resulting morphology. However, the mechanism that underlies the stabilisation of the liquid domains by the nanoparticles is not accurately captured by Equations (5) or (6). To address the observed discrepancies, a new model is required that properly reflects the properties and behavior of the nanoparticles during STrIPS.

### 3.6 A Theoretical Model for STrIPS Bijels

**FIGURE 7:**
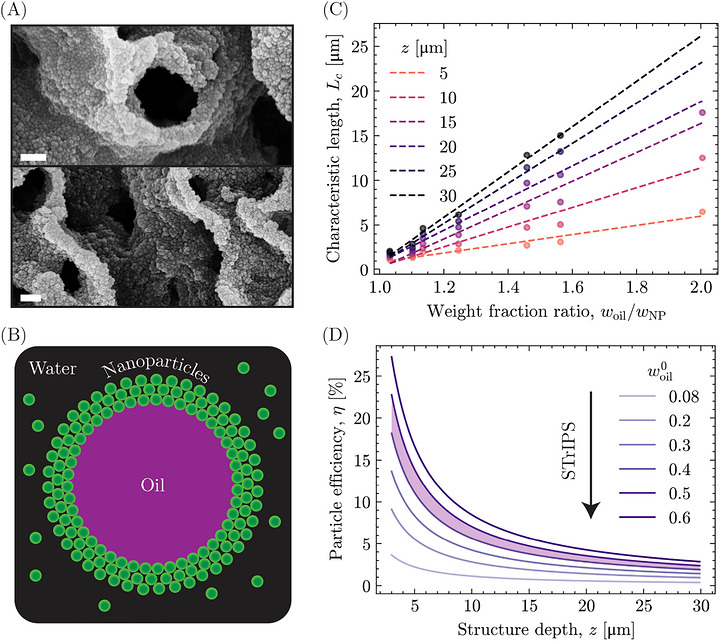
(A) SEM images of the nanoparticle scaffold surrounding the liquid domains in STrIPS bijels, revealing a multi‐layer structure. In each image the scalebar represents 200 nm. (B) Model system for relating the characteristic length $L_{c}$ to the nanoparticle weight fraction $w_{\mathrm{NP}}$ in STrIPS bijels. The system consists of spherical oil domains with radius $R_{0}$, stabilized by n‐layers of nanoparticles with diameter d. The characteristic length here taken as the diameter of the oil droplets $L_{c}=2R_{0}$. Only a fraction η of the nanoparticles are incorporated into the scaffold stabilizing the oil domains, the remainder being located in the ambient liquid phase. (C) The change in the characteristic length $L_{c}$, sampled at different depths z in STrIPS bijel films, for increasing weight fraction ratio $w_{\mathrm{oil}}/w_{\mathrm{NP}}$. A representative value of $w_{\mathrm{oil}}^{0}=0.43$, the weight fraction of the oil in the liquid phase, is used to calculate the ratio $w_{\mathrm{oil}}/w_{\mathrm{NP}}$. The dashed lines represent fits with Equation (10). (D) The particle efficiency η over the depth z in the STrIPS bijel film, determined from the slopes of lines fitted with Equation (10) as shown in (C). Increasing values of $w_{\mathrm{oil}}^{0}$ were used for these fits, representing progressive stages of STrIPS. The region associated with the structural arrest of STrIPS bijels is shaded purple.

The scaling between the characteristic length $L_{c}$ and the inverse volume fraction of the nanoparticles ${\tilde{\rho }}_{\mathrm{NP}}/w_{\mathrm{NP}}$ with Equation (6) relies on a few assumptions. For example, it considers a spherical geometry for the liquid domains. Domains in bijels are inherently non‐spherical, yet its proven efficacy for TIPS bijels indicates that this is not the core problem. Rather, the more pressing issue seems to be the assumption that stabilization occurs through particle monolayers, which incorporate all particles present in the system. As visible in Figure 7A, the nanoparticle scaffold of STrIPS bijels generally consists of multiple layers [42]. Moreover, the confocal images in Figure 4 indicate that a considerable fraction of the nanoparticles are not involved in the stabilization of the domains, instead residing in the bulk liquid phases. These two factors must be taken into account when trying to interpret the observed relationship between $L_{c}$ and $1/w_{\mathrm{NP}}$ for STrIPS bijels.

Figure 7B introduces an extended model system where both the formation of multi‐layers and the partial activity of the nanoparticles are included. This system consists of spherical oil domains of radius $R_{0}$, stabilized by n layers of close‐packed nanoparticles of diameter d. The diameter of the composite droplet $D_{n}$, twice the radius $R_{n}$, is then given by

(7) $$D_{n}=L_{c}+\left(2n-1\right)d,$$

where the diameter of the oil droplet $2R_{0}$ is taken as the characteristic length $L_{c}$. Not all of the nanoparticles are included in the scaffolds that cover the oil droplets. The particle efficiency η is here defined as the fraction of all nanoparticles present in the system that reside within the multi‐layer structure.

Under these conditions, with the full derivation provided in the ESI, the relationship between the characteristic length $L_{c}$ and the nanoparticle weight fraction $w_{\mathrm{NP}}$ becomes

(8) $$L_{c}=\frac{(2n-1)d}{\sqrt[{3}]{(1+\frac{3\sqrt{2}}{\pi }\frac{\eta \tilde{w}}{\tilde{\rho }}})-1},$$

where $\tilde{\rho }=\rho_{\mathrm{NP}}/\rho_{\mathrm{oil}}$ and $\tilde{w}=w_{\mathrm{NP}}/w_{\mathrm{oil}}$ respectively are the ratios of the densities and weight fractions of the oil and the nanoparticles. As evidenced in the ESI, for low values of η Equation (8) can be linearized to

(9) $$L_{c}\approx \frac{\pi \tilde{\rho }}{\sqrt{2}}\frac{(2n-1)d}{\eta \tilde{w}}.$$

Note that in contrast to the analogous Equation (5), Equation (9) includes the weight fraction ratio $\tilde{w}=w_{\mathrm{NP}}/w_{\mathrm{oil}}$ rather than just $w_{\mathrm{NP}}$. This is because for STrIPS bijels the content of the nanoparticles is sufficiently high that the weight fraction of either liquid phase cannot be considered independent, prohibiting a binary liquid simplification where $w_{\mathrm{oil}}\approx 1/2$ and thus $\tilde{w}\approx 2w_{\mathrm{NP}}$.

Forgoing such an approximation does have consequences for the application of Equation (9) to STrIPS bijels. In particular, the exchange of DEP and 1‐propanol with ambient toluene causes both the weight fraction of the oil $w_{\mathrm{oil}}$ and its density $\rho_{\mathrm{oil}}$ to change during the STrIPS process. Fortunately, an approximate measure of $w_{\mathrm{oil}}$ at the time of structural arrest can be deduced from the confocal images. In particular, confocal images with only the fluorescent signal of the oil phase, as shown in the top row of Figure 4, provide an average value of $w_{\mathrm{oil}}^{0}$, the weight fraction of the oil considering only the liquid components of the system. With a known nanoparticle loading $w_{\mathrm{NP}}$ accounting for the solid components, the combined weight fraction of all liquids in the system is then given by $1-w_{\mathrm{NP}}$. Accordingly, the weight fraction of the oil in the complete system follows from $w_{\mathrm{oil}}=w_{\mathrm{oil}}^{0}\left(1-w_{\mathrm{NP}}\right)$.

As shown in the ESI, confocal analysis of a representative STrIPS bijel film yields $w_{\mathrm{oil}}^{0}\approx 0.43$ at structural arrest, remarkably close to the value at the end of the compositional pathway in the phase diagram of Figure 1B. Taking into account the significant difference with the initial weight fraction $w_{\mathrm{DEP}}^{0}=0.08$ in the precursor, it can additionally be assumed that all DEP has been effectively replaced by ambient toluene at that point in the STrIPS process. Consequently, a value of $\tilde{\rho }=2.7$ is used for the density ratio in Equation (9) and its subsequent derivatives.

Taking the representative $w_{\mathrm{oil}}^{0}\approx 0.43$ to calculate the ratio $w_{\mathrm{oil}}/w_{\mathrm{NP}}$, the corresponding values of the characteristic length $L_{c}$ are plotted in Figure 7C. Again, $L_{c}$ is sampled at different depths z to reveal differences in behavior across the bijel structure. As in Figure 6C, the shape of the profiles in Figure 7C warrants the introduction of a maximum weight fraction ${\tilde{w}}_{max}$ as an empirical off‐set to Equation (9), resulting in

(10) $$L_{c}\approx \frac{\pi \tilde{\rho }}{\sqrt{2}}\frac{(2n-1)d}{\eta }\left(\frac{1}{\tilde{w}}-\frac{1}{{\tilde{w}}_{max}}\right).$$

Based on the SEM images shown in Figure 7A, in addition to numerous others [42], the number of layers in the nanoparticle scaffold of a STrIPS bijel is taken as the conservative estimate of n=3. In addition, the diameter of the individual nanoparticles is known to be 22 nm. Consequently, the profiles in Figure 7C can be fitted to Equation (10) to determine the final unknown: the particle efficiency η. The results of these fits are shown in Figure 7D, demonstrating a change in particle efficiency η with depth z in the bijel structure. The different profiles correspond to increasing liquid weight fractions of the oil phase $w_{\mathrm{oil}}^{0}$, representing the compositional change during the STrIPS process. The likely region of structural arrest for a STrIPS bijel, where $0.40\leq w_{\mathrm{oil}}^{0}\leq 0.50$, is shaded purple.

Regardless of the exact value of $w_{\mathrm{oil}}^{0}$, the results in Figure 7D indicate that the observed relationship between $L_{c}$ and $1/w_{\mathrm{NP}}$ for STrIPS bijels can be explained by a limited efficiency η of the nanoparticles. As already suggested by the images in Figure 3, only a relatively small fraction of the nanoparticles stabilize the liquid interface in the STrIPS bijel, with the remainder residing in the bulk phases. This fraction decreases with depth z in the bijel structure, which can be interpreted in at least two different ways.

The first is related to the functionalization of the nanoparticles with the surfactant CTAB. Hydrophobic functionalization is a transient property of the nanoparticles that depends on the local solvent concentration [14, 31, 43]. A rapid drop in the solubility of the surfactant, as occurs in the surface region of the bijel as a result of solvent depletion, can cause the functionalization of the nanoparticles to increase locally. These nanoparticles are comparatively more surface‐active than those in deeper regions of the bijel, where solvent levels remain higher. In turn, this corresponds to higher values for the particle efficiency η in the surface region.

The second interpretation is that a reduction in the particle efficiency with depth is inherent to the process of bijel formation via STrIPS. Namely, previous simulations of STrIPS show that the interplay between liquid phase separation and nanoparticle adsorption always causes the formation of larger domains deeper in the bijel, irrespective of the exact properties of the nanoparticles [28, 29]. Larger domains simply facilitate the adsorption of fewer nanoparticles, causing the particle efficiency to effectively decrease with depth.

In summary, a model system for particle‐stabilized emulsions is introduced that incorporates the formation of multi‐layer scaffolds, in addition to partial nanoparticle activity. By relating the characteristic length $L_{c}$ to the nanoparticle weight fraction $w_{\mathrm{NP}}$, the extended model is then used to deduce the general properties and behavior of nanoparticles in STrIPS bijel films. The observed morphological trends are found to be consistent with only a limited fraction of the nanoparticles stabilizing the liquid domains. This reduced efficiency of the nanoparticles, qualitatively supported by confocal images, further decreases with depth into the bijel structure.

## 4 Discussions

The results of this work demonstrate the notable influence of the nanoparticle loading on the structure of STrIPS bijels, embedding observed morphological trends in a general context of nanoparticle behavior. However, some topics remain that warrant further discussion. These topics can be loosely clustered around the two main classes of bijel components: nanoparticles and liquids.

### 4.1 Nanoparticles

Central to the findings involving the nanoparticles is the role of the surfactant CTAB. By controlling the stability of the nanoparticles in the precursor mixture, the concentration of the surfactant effectively determines the nanoparticle content in the resulting bijels. However, CTAB achieves this by modifying the surface properties of the nanoparticles, which also influences their self‐assembly dynamics [31] and their overall behavior during STrIPS [13, 14].

As demonstrated in Figure 4, the concentration of CTAB governs the distribution of the nanoparticles in the bijel structure, with more hydrophobic nanoparticles transitioning into the oil‐rich channels at higher concentrations. The partitioning of nanoparticles between the different channels is especially relevant, as the local environment can change their aggregation behavior and therefore their potential effectiveness in stabilizing liquid domains [13]. Notably, the latter effect is not taken into account by the model in Section 3.6, which assumes constant effectiveness of the nanoparticles across weight fractions.

Consequently, to independently investigate the morphological influence of the nanoparticles, it is key to decouple the role of the surfactant. Although this could be achieved in an alternative STrIPS system without a direct connection between nanoparticle loading and surfactant concentration, future work ideally aims to reproduce the findings presented here in a system that does not contain a surfactant at all. For example, with silica nanoparticles that have been rendered surface‐active through silane functionalization instead [38].

Furthermore, there appears to be a discrepancy with previous work regarding the stability of nanoparticles in the bijel precursor, as presented in Figure 2. There, an increase in the concentration of the surfactant was also linked to enhanced stability of the nanoparticles, yet at low concentrations the precursor destabilized through liquid phase separation rather than aggregation [27]. This difference can be explained by the higher solvent content of the precursor mixture in this work. When the composition of the precursor is located very close to the binodal, as in the previous work, the hydrophilic nature of the silica surface at a low surfactant concentration can induce liquid phase separation to lower the energy of the overall system. However, here the solvent content is sufficiently high to keep the precursor miscible even at low concentrations of surfactant. With phase separation prevented, the precursor instead destabilizes through the aggregation of the hydrophilic nanoparticles.

Finally, although the domain size profiles in Figure 5C show the evident influence of nanoparticle loading on STrIPS bijels, a prominent feature is not mentioned. Namely, the total thickness of the coated bijel film appears to increase with higher weight fractions of the nanoparticles. As such, the maximum domain size cannot be used as a comparative metric for structural analysis, leaving only the slope of the profiles. The production of uniform bijel films could solve this issue, which would also help to find the exact relationship between the film thickness and the nanoparticle content. However, the vertical dip‐coating method employed in this work is not suitable for this purpose, as it inherently produces a gradient in the film thickness due to gravity. While outside the scope of this work, a potential remedy is to produce bijel films of controllable, uniform thickness through slot‐die coating instead [23].

### 4.2 Liquids

The mass transport of liquid components is a core part of the STrIPS process, yet is generally presented in a simplified, albeit functional, manner. In particular, it is often considered solely from the perspective of the diffusing solvent, while the influx of the ambient oil is just as relevant. For example, the ideal functionalization of the nanoparticles to stabilize liquid domains is determined by the volume ratio of the oil and aqueous phases in the bijel [44]. In addition, the extended theoretical model introduced in Section 3.6 also requires input on the local liquid composition. However, during STrIPS the liquid composition, and by extension the ratio of oil and aqueous phases, varies with both time and depth in the bijel film, adding another layer of complexity for the interpretation of experimental findings.

In this study, these factors are addressed through a set of approximations regarding mass transfer during STrIPS. Although acceptable as a first step, it is clear that the associated exchange of the solvent and oils has to be elucidated more extensively to gain further control over the structure of the resulting bijel films. It would be especially worthwhile to spatially resolve the local liquid composition across the bijel film at different stages of the STrIPS process, which could be achieved by extending existing frameworks of numerical simulations [28, 29] supported by experiments.

## 5 Conclusions

This work elucidates the influence of the nanoparticle content on the morphology of bijels produced via STrIPS. By selectively tuning the colloidal stability through surfactant functionalization, bijel precursors with varying weight fractions of nanoparticles were prepared. Supported bijel films were fabricated with these precursors, followed by an investigation of their structure with confocal microscopy. Spectral analysis of confocal images enabled the morphology of the entire bijel film to be resolved, yielding depth profiles of the average size of the liquid domains.

Higher nanoparticle loadings result in a smaller overall size of the liquid domains, in agreement with numerical simulations, in addition to a lower gradient over the bijel film. Through quantitative analysis, an empirical equation is established that relates the domain size at a certain depth to the weight fraction of the nanoparticles. Finally, a new model system is introduced that better captures the behavior of the nanoparticles in STrIPS bijels. Here, the observed morphological scaling between the domain size and nanoparticle loading is ascribed to a limited particle efficiency.

## Author Contributions

**Jesse M. Steenhoff**: conceptualization, formal analysis, investigation, methodology, software, validation, visualization, writing – original draft, writing – review & editing. **Martin F. Haase**: conceptualization, funding acquisition, supervision, writing – review & editing.

## Conflicts of Interest

The authors declare no conflicts of interest.

## Supporting information


**Supporting File**: advs76965‐sup‐0001‐SuppMat.pdf.

## Data Availability

The data that support the findings of this study are available from the corresponding author upon reasonable request.
