# Supplementary material for: Structural Control Over Bicontinuous Emulsions from Solvent‐Transfer Induced Phase Separation Through Nanoparticle Loading
Source: Adv Sci (Weinh). 2026 Aug 13:e76965. Online ahead of print. doi: 10.1002/advs.76965 (PMC13472128; doi:10.1002/advs.76965)
Supplement: Supplementary file 1 [file ADVS-9999-e76965-s001.pdf]

Electronic Supplementary Information (ESI) for : "Structural Control over Bicontinuous Emulsions from Solvent-Transfer Induced Phase Separation through Nanoparticle Loading"

Jesse M. Steenhoff*[1] and Martin F. Haase**[1]

Van 't Hoff Laboratory for Physical and Colloid Chemistry, Utrecht University, Utrecht, The Netherlands

(**m.f.haase@uu.nl)

(*j.m.steenhoff@uu.nl)

(Dated: 8 July 2026)

The ESI provides additional information to support the findings of the main document. Its content is divided as follows: Section I covers the preparation of the precursor mixture for a STrIPS bijel, which includes a detailed example for a 1 mL sample with varying nanoparticle loadings. Next, Section II provides some more context on the compositional pathway shown in the phase diagram of the precursor mixture, in particular the explicit calculation and underlying assumptions. In Section III, the interactions between the surfactant and nanoparticles are characterised, specifically by measuring the distributions in the electrophoretic mobility and hydrodynamic diameter of the nanoparticle dispersions at different concentrations of the surfactant. Section IV further elucidates the extended model system for particle-stabilised emulsions introduced in this work. In particular, it contains the full derivation of the expression that relates the characteristic length L_c to the nanoparticle loading w_{NP} when accounting for the formation of multi-layer structures and limited particle efficiency. Finally, Section V describes the procedure for calculating liquid weight fractions from confocal images of STrIPS bijel films, providing the input data for some of the analysis performed in this work.

I. BIJEL PRECURSORS FOR STRIPS

The precursor used for the fabrication of STrIPS bijels is a relatively complex mixture that contains both liquids and solid components. The principal liquid components are water and diethyl phthalate (DEP), which are immiscible by themselves, in addition to the solvent 1-propanol. It also contains glycerol, which serves to match the refractive index of the aqueous phase in the final bijel with the hexane-based image medium for confocal microscopy. The solid components are primarily Ludox TMA silica nanoparticles (batch number #1003481587), stabilised in the precursor with the surfactant hexadecyltrimethylammonium bromide (CTAB).

The following is an example preparation of 1 mL of the STrIPS bijel precursor with a nanoparticle loading of $w_{\mathrm{NP}} = 0.29$. These steps require a 50 w% dispersion of non-acidified Ludox TMA nanoparticles in water, which can be prepared according to the descriptions in the Methods section of the main document. Additionally, different stock solutions of 200 mM CTAB and 50 w% glycerol, both in 1-propanol, are needed.

First, the organic phase of the precursor is prepared by mixing 68 mg of DEP, 120 mg of the CTAB/1-propanol solution, 208 mg of the glycerol/1-propanol solution and 77 mg of pure 1-propanol. This mixture is then thoroughly homogenised with a vortex mixer. Subsequently, the pH of the concentrated nanoparticle dispersion is reduced to a value of 1.8 by adding 1 M HCl under continuous stirring. Immediately after this acidification step, 680 mg of the nanoparticle dispersion is added to the organic phase of the precursor. Following homogenisation with a vortex mixer, this precursor is then centrifuged at 16 160 ×g for 30 min (Microfuge 16, Beckman Coulter). In case of nanoparticle aggregation, the supernatant is taken as the final STrIPS precursor. However, for this particular composition, very little to no nanoparticle aggregation should be present.

These steps result in a precursor of the following composition: $w_{\mathrm{DEP}} = 0.06$, $w_{\mathrm{1prop}} = 0.26$, $w_{\mathrm{wat}} = 0.29$, $w_{\mathrm{gly}} = 0.09$, and $w_{\mathrm{NP}} = 0.29$. Under the assumption that glycerol fully resides in the water domains after phase separation, a combined aqueous phase can be constructed which then has a total weight fraction of $w_{\mathrm{aq}} = w_{\mathrm{wat}} + w_{\mathrm{gly}} = 0.38$. Furthermore, since the phase behaviour of the precursor mixture is dominated by the liquid-liquid phase separation, it is best understood by considering the relative composition of only the liquid phase. The total contribution of the liquids to the precursor is $1 - w_{\mathrm{NP}} = 0.71$, such that the weight fractions of the individual liquids in the isolated liquid phase follow from $w^0 = w/(1 - w_{\mathrm{NP}})$. As presented in the phase diagram of Figure 1B of the main document, this then yields a composition of $w^0_{\mathrm{DEP}} = 0.08$, $w^0_{\mathrm{1prop}} = 0.37$,

	c [mM]					
	15	19	24	28	34	38
$\Delta m_{\mathrm{CTAB/1prop}}$ [mg]	60	75	98	113	135	150
$\Delta m_{\mathrm{1prop}}$ [mg]	137	122	100	85	62	47
w_{NP} [%]	17.6	21.6	27.5	29.2	28.9	27.9

TABLE S1. Amounts of 20 mM CTAB/1-propanol stock solution ($\Delta m_{\mathrm{CTAB/1prop}}$) and pure 1-propanol ($\Delta m_{\mathrm{1prop}}$) required to prepare 1 mL of a precursor mixture with a certain CTAB concentration (c). Also listed are the associated nanoparticle loadings (w_{NP}) of these precursors.

$w^0_{\mathrm{aq}} = 0.55$ for the liquid phase of the precursor mixture.

Finally, the concentration of CTAB in the precursor described above is 30 mM. This considers the full volume of the mixture, including the nanoparticles. According to Figure 2A of the main document, 30 mM is optimal to stabilise nearly all nanoparticles initially added with the aqueous dispersion. The concentration of CTAB can be varied by changing only the relative amounts of the 200 mM CTAB/1-propanol stock solution and pure 1-propanol while preparing the organic phase of the precursor. However, note that this will also change the extent of nanoparticle aggregation and therefore the loading present in the final precursor after centrifugation. Table S1 provides an overview of the amounts of CTAB/1-propanol and pure 1-propanol required to prepare 1 mL of some selected precursor mixtures with varying CTAB concentration used in this work, along with the associated nanoparticle loading.

II. COMPOSITIONAL PATHWAY OF BIJEL PRECURSORS DURING STRIPS

The phase diagram in Figure 1B of the main document shows a pathway that represents the compositional change of the precursor mixture when both its solvent (1-propanol) and initial oil (DEP) are exhanged with oil from the ambient liquid phase (toluene). As discussed in detail, this is by no means meant to accurately capture the complex process of mass transfer that occurs during STrIPS. However, it can provide some qualitative insight into the general direction of compositional change during the very early stages of the process.

The calculation underlying the compositional pathway is predicated on the assumption that the influx of toluene from the ambient phase fully compensates for the loss of 1-propanol and DEP from the precursor. Treating the total volume of the bijel precursor as constant, this thus implies

that

$$\Delta V_{\mathrm{tol}} = -\Delta V_{\mathrm{1prop}} - \Delta V_{\mathrm{DEP}}, \tag{1}$$

with ΔV_{tol}, $\Delta V_{\mathrm{1prop}}$ and ΔV_{DEP} as the exchanged volumes of toluene, 1-propanol, and DEP, respectively. Eq. (1) can alternatively be expressed in terms of the exchanged masses Δm_{tol}, $\Delta m_{\mathrm{1prop}}$ and Δm_{DEP}, which then takes the form of

$$\Delta m_{\mathrm{tol}} = -\frac{\rho_{\mathrm{tol}}}{\rho_{\mathrm{1prop}}}\Delta m_{\mathrm{1prop}} - \frac{\rho_{\mathrm{tol}}}{\rho_{\mathrm{DEP}}}\Delta m_{\mathrm{DEP}} \tag{2}$$

where ρ_{tol}, ρ_{1prop} and ρ_{DEP} are the respective densities of toluene, 1-propanol, and DEP. The exchanged mass Δm of any component in the precursor mixture can in turn be written in terms of its weight fraction w and the total precursor mass m_T, namely $\Delta m = m_T \Delta w + w \Delta m_T$. Applying this to Eq. (2) subsequently yields

$$\Delta w_{\mathrm{tol}} + w_{\mathrm{tol}}\frac{\Delta m_T}{m_T} = -\frac{\rho_{\mathrm{tol}}}{\rho_{\mathrm{1prop}}}\left(\Delta w_{\mathrm{1prop}} + w_{\mathrm{1prop}}\frac{\Delta m_T}{m_T}\right) - \frac{\rho_{\mathrm{tol}}}{\rho_{\mathrm{DEP}}}\left(\Delta w_{\mathrm{DEP}} + w_{\mathrm{DEP}}\frac{\Delta m_T}{m_T}\right) \tag{3}$$

By assuming that $w\frac{\Delta m_T}{m_T} \ll \Delta w$, which is not unreasonable for the exchange of liquids with relatively comparable densities, Eq. (3) can be simplified into

$$\Delta w_{\mathrm{tol}} \approx -\frac{\rho_{\mathrm{tol}}}{\rho_{\mathrm{1prop}}}\Delta w_{\mathrm{1prop}} - \frac{\rho_{\mathrm{tol}}}{\rho_{\mathrm{DEP}}}\Delta w_{\mathrm{DEP}}. \tag{4}$$

With an initial composition of $w^0_{\mathrm{DEP}} = 0.08$, $w^0_{\mathrm{1prop}} = 0.37$, $w^0_{\mathrm{aq}} = 0.55$, in addition to the density ratios $\rho_{\mathrm{tol}}/\rho_{\mathrm{1prop}} = 1.07$ and $\rho_{\mathrm{tol}}/\rho_{\mathrm{DEP}} = 0.77$, Eq. (4) then provides a final composition of $w^0_{\mathrm{tol}} = 0.46$, $w^0_{\mathrm{aq}} = 0.54$ after complete removal of 1-propanol and DEP from the precursor. Tracing a line from the initial to this final composition in the phase diagram, the precursor crosses the binodal curve very close to the critical point. Consequently, the compositional pathway indicates that phase separation during STrIPS is likely to occur through spinodal decomposition, rather than nucleation and growth, for the chosen initial composition of the precursor mixture.

III. CHARACTERISATION OF NANOPARTICLE-SURFACTANT INTERACTIONS

To provide additional context regarding the stability of the nanoparticles in the precursor mixture, as discussed in Section III B of the main document, the interactions between the surfactant (CTAB) and the silica nanoparticles in different liquid media warrant particular attention. Our current hypothesis is that the trend in the stability of the nanoparticles visible in Figure 2A of

the main document can be explained in terms of the relative interactions between the surfaces of individual nanoparticles and the liquid medium.

Specifically, since bare silica nanoparticles have a rather polar surface, a certain degree of surface functionalisation by the apolar surfactant tails is required to stabilise them in the relatively apolar liquid medium of the precursor mixture. Initially, increasing the concentration of the surfactant thus stabilises a larger fraction of the silica nanoparticles, resulting in higher nanoparticle loadings in the bijel precursor. However, above a threshold surfactant concentration, roughly 30 mM for the precursor mixture in this work, the hydrophobic interactions between the surfactant-functionalised nanoparticles become sufficiently strong to again induce aggregation, which in turn lowers the content of the nanoparticles in the final bijel precursor.

A key factor in this mechanism of stabilisation is the strong influence of the surface charge distribution among the different nanoparticles. The surface charge governs the extent of the interaction between the silica nanoparticles and the cationic surfactant, so that a distribution in the surface charge should give rise to a distribution in the surface functionalisation. The surface charge distributions of the silica nanoparticles used in this study (Ludox TMA, #1003481587) were characterised by measuring the electrophoretic mobilities of aqueous dispersions by laser Doppler electrophoresis (Malvern Zetasizer Pro). The results are shown in Figure S1 of this document for different pH values of the aqueous dispersion.

At pH 6, deprotonated silanol groups are the main contributors to the overall surface charge of the nanoparticles. These deprotonated silanols impart a negative charge to the surface of the nanoparticles, yielding a sharp distribution with a strongly negative average of $-4.1(1) \times 10^{-8}\,\mathrm{m}^2/(\mathrm{Vs})$ for the electrophoretic mobility. Decreasing the pH to 4 again protonates some of the deprotonated silanols, thus neutralising part of the negative surface charge and shifting the average electrophoretic mobility to a more positive value of $-3.2(1) \times 10^{-8}\,\mathrm{m}^2/(\mathrm{Vs})$. Going further down in pH to 2, around the isoelectric point of silica nanoparticles[1], the fast majority of surface silanols should exist in their neutral state (or protonated state, reasoning from an initially deprotonated starting point)

However, the electrophoretic mobility distribution at pH 2 retains a distinctly negative average of $-1.0(1) \times 10^{-8}\,\mathrm{m}^2/(\mathrm{Vs})$, indicating that a considerable fraction of nanoparticles still carry a negative charge. For Ludox TMA nanoparticles, this negative charge originates from aluminium substitutions in the silica framework, which provide a persistent negative lattice charge even at low pH[2]. As such, the broadening of the electrophoretic mobility distribution at pH 2 could be related

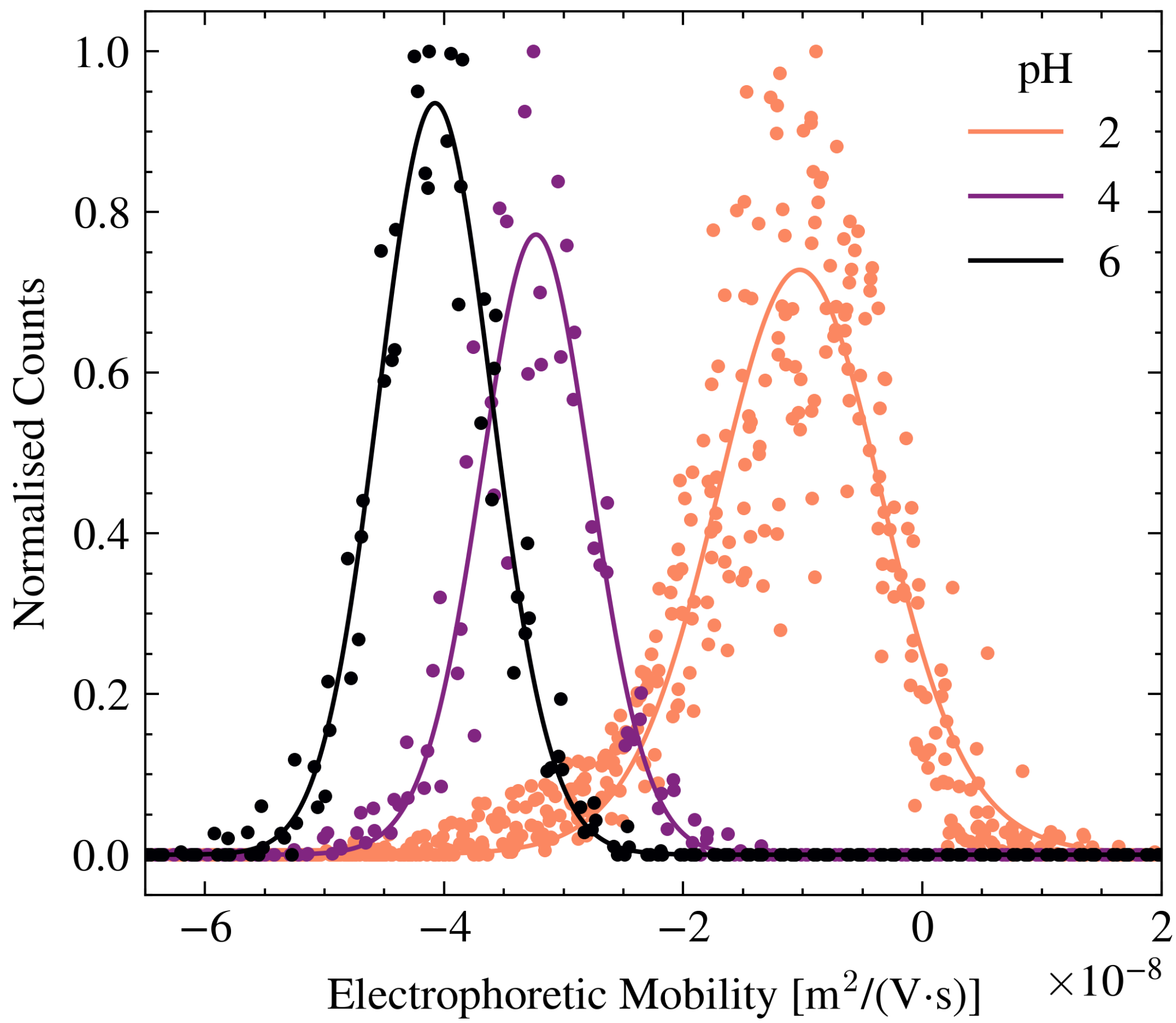

FIG. S1. Electrophoretic mobility distributions of Ludox TMA silica nanoparticles in 1 w% aqueous dispersions with varying pH, measured by laser Doppler electrophoresis. The solid lines are the averaged Gaussian fits of at least three individual measurements.

to a different aluminium content between individual nanoparticles, which might exhibit greater variability than the average density of silanol groups relevant at higher pH.

Irrespective of the exact origin, Figure S1 shows that for the low pH experimental conditions used in this study, the nanoparticles still display an evident distribution in their electrophoretic mobilities. Accordingly, different nanoparticles in the dispersion will have different extents of interactions with the cationic surfactant CTAB. To further clarify this behaviour, aqueous 1 w% dispersions of nanoparticles were prepared at pH 2 with CTAB concentrations that varied between 5 μM and 250 μM. A selection of these dispersions is shown in Figure S2A of this document, roughly 24 h after sample homogenisation through vortex mixing. The dispersions become increasingly turbid with higher concentrations of CTAB, indicating that the nanoparticles interact with the surfactant and undergo limited aggregation in the aqueous medium as a result of hy-

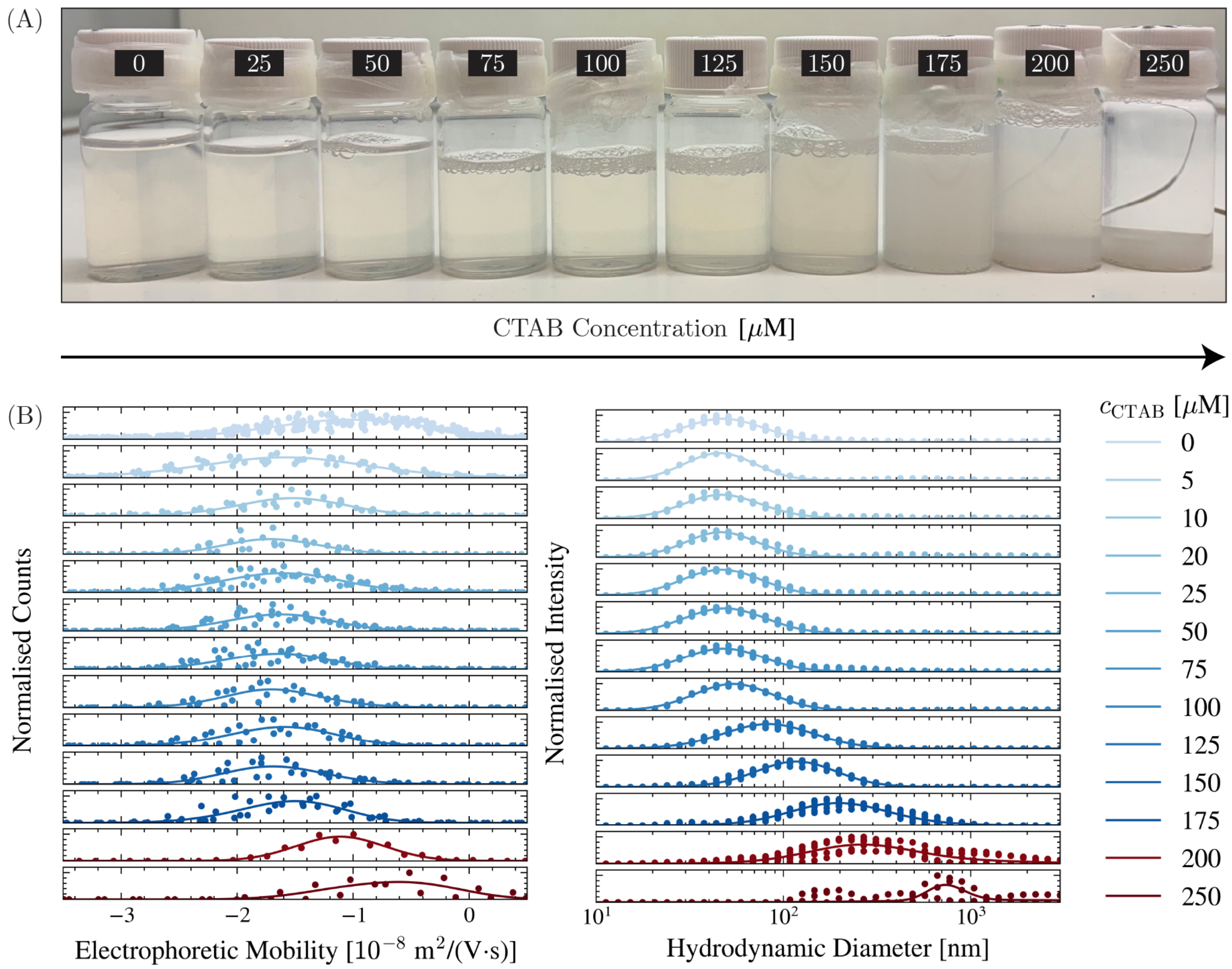

FIG. S2. (A) Selected nanoparticle dispersions containing 1 w% Ludox TMA at pH 2 with varying concentrations of the cationic surfactant CTAB. (B) Distributions of the electrophoretic mobility (left) and hydrodynamic diameter (right) of the Ludox TMA nanoparticle dispersions with increasing CTAB concentration. The distributions shown in red correspond to the nanoparticles in supernatant, isolated from the samples that formed a distinct sediment layer at the two highest concentrations of CTAB. The solid lines are the averaged Gaussian fits of at least three individual measurements.

drophobic forces.

Regardless, the nanoparticles remain dispersed at CTAB concentrations below 200 μM, with a sediment layer forming only for the samples with concentrations of 200 μM and 250 μM. For these samples, only part of the nanoparticles end up in the sediment layer, which is particularly clear for the 200 μM sample, the remainder staying dispersed in the supernatant. This observation is fully in line with a variation in the degree of surfactant functionalisation for the nanoparticles due to the

distribution in their surface charge.

Subsequently, both the distributions in the electrophoretic mobility and hydrodynamic diameter of the nanoparticles were measured through laser Doppler electrophoresis and dynamic light scattering, respectively. For the two samples with a sediment layer, only the nanoparticles present in the supernatant were measured. The results are collected in Figure S2B, showing the evolution of the distributions with increasing concentrations of CTAB. The measurements for the hydrodynamic diameter match the observations in Figure S2A, showing a progressive increase in size as a result of the surfactant-induced aggregation of the nanoparticles, which becomes particularly prominent for CTAB concentrations above 100 μM. Interestingly, this trend is not equally apparent from the measurements of the electrophoretic mobility.

At first glance, the interaction between a negatively charged surface and a cationic surfactant is expected to yield a lower magnitude of the electrophoretic mobility due to partial neutralisation of the charges present. However, the distributions of the electrophoretic mobility in Figure S2B do not directly reflect this behaviour. Rather, the initial introduction of 5 μM cationic CTAB causes the average electrophoretic mobility of the nanoparticles to become even more negative. As this is also accompanied by a distinct sharpening of the distribution, this counter-intuitive behaviour could be explained by a filtering of the more weakly charged nanoparticles, which are already inherently less stable against aggregation. If the CTAB, perhaps transiently, destabilises these nanoparticles, the distribution in the electrophoretic mobility undergoes an apparent shift to more negative values. However, the aggregation of nanoparticles associated with this explanation is not readily evident from the complementary measurements of the hydrodynamic diameter.

In addition, increasing the surfactant concentration from 5 μM to 175 μM does not seem to significantly influence the distribution of the electrophoretic mobility for the nanoparticles. Only for the nanoparticles in the supernatant samples, where initial aggregation is thus already sufficiently strong to form a sediment, does the electrophoretic mobility shift to slightly more positive values. This behaviour could be explained by considering that for Ludox TMA at pH 2, the cationic surfactant primarily interacts with the more delocalised lattice charge caused by the aluminium substitutions, rather than directly with a deprotonated surface silanol. The different nature of the negative surface charge for Ludox TMA could make it more difficult to directly neutralise, or perhaps make it more akin to an ion-exchange where the cationic surfactant replaces the counter-ion inherently associated with the aluminium lattice charge. If this interaction places the initial counter-ion outside the slipping plane of the electric double layer, the electrophoretic mobility

would not be as affected by the attachment of the surfactant.

However, these different explanations all remain very speculative. Clearly, additional research is required to resolve the specifics of the interactions between the surfaces of Ludox TMA nanoparticles and the cationic surfactant CTAB, yet this is outside the scope of the current work. In particular, repeating the experiments in Figure S2 using the liquid medium of the precursor mixture rather than water would be preferable, since it would better match the conditions of the STrIPS bijel. At present, these types of measurements are not included as they would require a more extensive characterisation of the precursor properties, such as the viscosity and dielectric constant, than was feasible for this work.

IV. EXTENDED MODEL SYSTEM FOR PARTICLE-STABILISED EMULSIONS

The main document introduces an extended model system for relating the characteristic length L_c to the nanoparticle weight fraction w_{NP} in particle-stabilised emulsions. The extended model explicitly takes into account the formation of nanoparticle multi-layers, in addition to the option that not all nanoparticles in the system are included in this scaffold. The model system, illustrated in Figure S3 of this document, consists of spherical oil droplets of radius R_0 stabilised by n layers of attached nanoparticles of diameter d. The diameter of these composite droplets, twice the radius R_n, is given by

$$D_n = L_c + (2n-1)d, \tag{5}$$

where the diameter of the bare oil droplet $2R_0$ is taken as the characteristic length L_c.

With this system established, the first step is to quantify the total number of oil droplets and nanoparticles present. For a total volume V_{oil} of the oil phase, the system includes $6V_{\mathrm{oil}}/\pi L_c^3$ particle-decorated droplets. Similarly, a total particle volume V_{NP} provides $6V_{\mathrm{NP}}/\pi d^3$ individual nanoparticles to the system. Not all of these nanoparticles are incorporated into the nanoparticle scaffolds covering the droplets, with the fraction of incorporated particles relative to the total number being defined as the particle efficiency η. Consequently, the number of particles that make up the nanoparticle shells in the system is given by $6\eta V_{\mathrm{NP}}/\pi d^3$. At the same time, this number is also equivalent to the total number of oil droplets (one shell per droplet) times the number of nanoparticles included in each shell N_{shell}, such that

$$\frac{\eta V_{\mathrm{NP}}}{d^3} = \frac{V_{\mathrm{oil}}}{L_c^3} N_{\mathrm{shell}}. \tag{6}$$

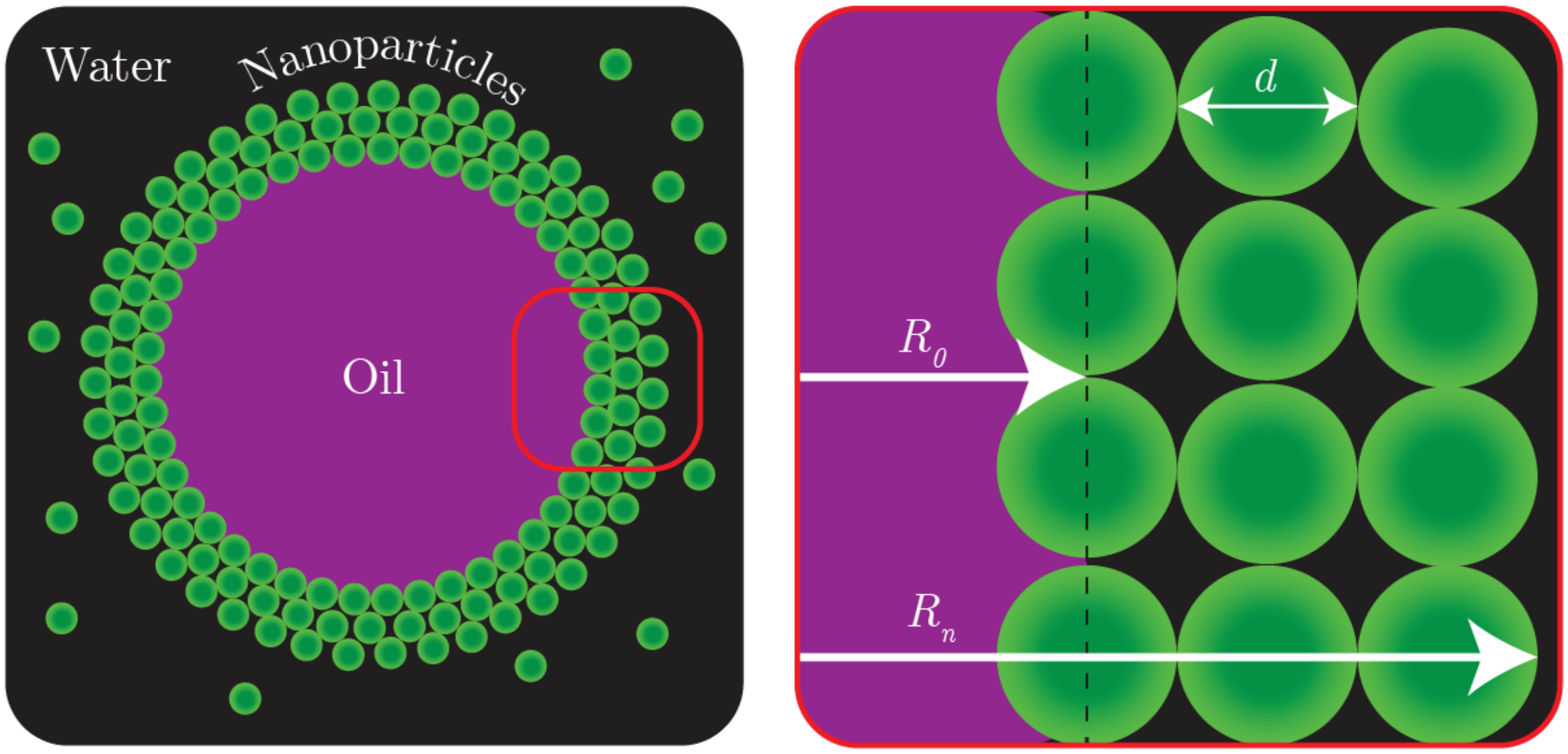

FIG. S3. Schematic illustration of the extended model system for a particle-stabilised emulsion. It consists of spherical oil domains of radius R_0, which are covered by n layers of nanoparticles of diameter d. These composite droplets then have a radius R_n, explicitly including the contributions from the nanoparticle scaffold. Not all nanoparticles in the system reside in these particle shells, with the fraction of incorporated particles given by the particle efficiency η.

To calculate the number of nanoparticles per shell, consider that the total volume of each nanoparticle shell is given by

$$V_{\text{shell}} = \frac{\pi}{6}\left(D_n^3 - L_c^3\right). \tag{7}$$

By assuming a close-packing of the nanoparticles, with a packing fraction $\pi/(3\sqrt{2})$, the total volume of the nanoparticles per shell then becomes

$$V_{\text{shell}}^* = \frac{\pi^2}{18\sqrt{2}}\left(D_n^3 - L_c^3\right). \tag{8}$$

Dividing the available shell volume V_{shell}^* by the volume of an individual nanoparticle then yields the number of nanoparticles per shell

$$N_{\text{shell}} = \frac{\pi}{3d^3\sqrt{2}}\left(D_n^3 - L_c^3\right). \tag{9}$$

This expression can then be substituted in Eq. (6) to give

$$\eta\frac{V_{\text{NP}}}{V_{\text{oil}}} = \frac{\pi}{3\sqrt{2}}\left(\frac{D_n^3}{L_c^3} - 1\right), \tag{10}$$

which can then be rearranged to isolate the ratio D_n/L_c as

$$\frac{D_n}{L_c} = \sqrt[3]{1 + \frac{3\eta\sqrt{2}}{\pi}\frac{V_{\mathrm{NP}}}{V_{\mathrm{oil}}}}. \tag{11}$$

Subsequently, Eq. (5) can be used to explicitly incorporate the dependence of this ratio D_n/L_c on the number of nanoparticle layers n. The result is an expression that relates the characteristic length L_c to the particle efficiency η, the number of nanoparticle layers n, and the volume ratio $V_{\mathrm{NP}}/V_{\mathrm{oil}}$ as

$$L_c = \frac{(2n-1)d}{\sqrt[3]{1 + \frac{3\eta\sqrt{2}}{\pi}\frac{V_{\mathrm{NP}}}{V_{\mathrm{oil}}}} - 1}. \tag{12}$$

The volume ratio $V_{\mathrm{NP}}/V_{\mathrm{oil}}$ in Eq. (12) can be converted into a weight fraction ratio, which then yields

$$L_c = \frac{(2n-1)d}{\sqrt[3]{1 + \frac{3\sqrt{2}}{\pi}\frac{\eta\tilde{w}}{\tilde{\rho}}} - 1}, \tag{13}$$

where $\tilde{\rho} = \rho_{\mathrm{NP}}/\rho_{\mathrm{oil}}$ and $\tilde{w} = w_{\mathrm{NP}}/w_{\mathrm{oil}}$ are the ratios of the densities and weight fractions of the oil and the nanoparticles, respectively. Although Eq. (13) already achieves the intended purpose of relating the characteristic length L_c to the weight fraction of the nanoparticles w_{NP}, it can be further simplified by applying a truncated Taylor expansion of the cube root: $\sqrt[3]{1+x} \approx 1 + x/3$ for $x \ll 1$. This approximation, which becomes increasingly accurate at lower values of the particle efficiency η, finally results in

$$L_c \approx \frac{\pi\tilde{\rho}}{\sqrt{2}}\frac{(2n-1)d}{\eta\tilde{w}}, \tag{14}$$

that is then used to fit the profiles in Figure 7 of the main document.

V. LIQUID WEIGHT FRACTIONS FROM CONFOCAL IMAGES

The analysis shown in Figure 7 of the main document involves the weight fraction ratio $w_{\mathrm{oil}}/w_{\mathrm{NP}}$ of oil and nanoparticles in the precursor. Because the solvent in the precursor mixture is gradually replaced by oil from the ambient phase, this ratio actually changes during STrIPS. Currently, there are no methods to experimentally determine $w_{\mathrm{oil}}/w_{\mathrm{NP}}$ *in situ*, yet confocal images of the final bijel structure can provide an estimation at the moment of structural arrest. In particular, these confocal images allow for an approximate measure of w^0_{oil}, which is the weight fraction of the oil considering only the liquid components of the system.

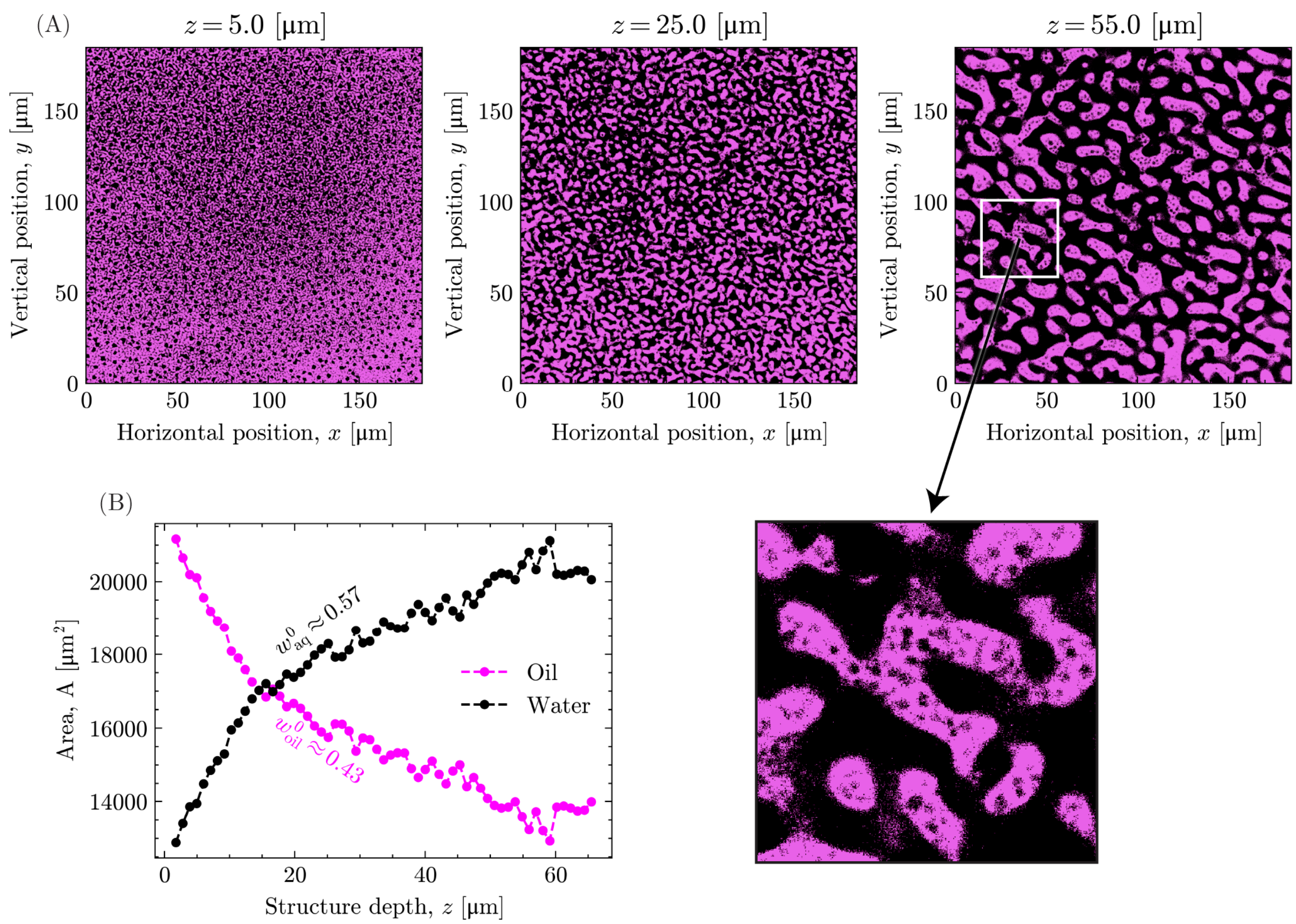

FIG. S4. (A) Confocal cross-sections at different depths z in a representative STrIPS bijel film, containing 27 w% nanoparticles. Only the binarised signal from the oil channels is shown here. The zoomed-in region of the final image shows the presence of small, nucleated structures of the aqueous phase in the channels of the oil phase. (B) Profiles of the cross-sectional area A for the oil and aqueous phases, taken over the depth z of the bijel film. The weight fraction of the oil in the total liquid phase w^0_{oil} is calculated by integrating these depth profiles according to Eq. (15), which here results in the shown value of $w^0_{\text{oil}} \approx 0.43$.

The process of determining w^0_{oil} from confocal images of STrIPS bijel films is demonstrated in Figure S4. By binarising the signal from the oil channels, shown in Figure S4A, each confocal image provides the cross-sectional area A_{oil} of the oil phase at a specific depth z in the bijel film. Since the total area of the image is constant, this also yields the cross-sectional area A_{aq} of the aqueous phase. The results of different confocal images can then be combined to create profiles of the cross-sectional area over the depth of the bijel film, as shown in Figure S4B for both phases.

Interestingly, these depth profiles indicate an inversion of the relative prominence of the oil and water phases. The oil phase occupies a larger fraction of the available area close to the surface of

the bijel film, yet this gradually decreases with depth until the water phase eventually takes over. The surface region of the bijel generally experiences a larger influx of oil from the ambient phase, so that the transition could be a natural consequence of mass transfer during STrIPS. However, it also has to be considered that the signal from the oil channels can deteriorate due to slight mismatches in the refractive index, which becomes more pronounced deeper in the bijel film.

Finally, the last image of Figure S4A reveals the appearance of nucleated structures within the oil phase deep in the bijel film. They are highlighted in the zoomed-in region of the image, showing distinct nuclei of the aqueous phase in the oil channels. Not only do these nuclei reduce the effective cross-sectional area measured for the oil phase, their high-frequency contributions to k-space can somewhat distort the structural analysis as presented in Figure 5 of the main document.

Irrespective of these observations, the main purpose of the profiles in Figure S4B is to calculate the value of w^0_{oil}. The volume of either phase is simply given by the integral of the respective depth profile $V = \int A\,dz$, such that the weight fraction of the oil in the pure liquid phase w^0_{oil} is given by

$$w^0_{\mathrm{oil}} = \frac{\rho_{\mathrm{oil}} \int A_{\mathrm{oil}}\,dz}{\rho_{\mathrm{oil}} \int A_{\mathrm{oil}}\,dz + \rho_{\mathrm{aq}} \int A_{\mathrm{aq}}\,dz}, \tag{15}$$

with ρ_{oil} and ρ_{aq} the densities of the oil and aqueous phases, respectively. For the representative profiles shown in Figure S4, Eq. (15) yields the value of $w^0_{\mathrm{oil}} \approx 0.43$ with densities of $\rho_{\mathrm{aq}} = 0.998$ g/mL and $\rho_{\mathrm{oil}} = 0.862$ g/mL for water and toluene, respectively.

With an experimentally determined value of w^0_{oil}, the ratio $w_{\mathrm{oil}}/w_{\mathrm{NP}}$ can be calculated assuming that the total mass of the bijel changes very little during STrIPS. That is, the mass of the solvent leaving the system is roughly offset by the mass of the oil flowing in. In that case, the weight fraction of the nanoparticles w_{NP} remains constant, such that the weight fraction of the total liquid phase is given by $1 - w_{\mathrm{NP}}$ at any point during STrIPS. Finally, the weight fraction of the oil in the total precursor follows directly from $w_{\mathrm{oil}} = w^0_{\mathrm{oil}}(1 - w_{\mathrm{NP}})$.

REFERENCES

[1]Z. G. Cui, L. L. Yang, Y. Z. Cui, and B. P. Binks, Langmuir **26**, 4717–4724 (2010).

[2]A. J. Sprockel, M. A. Khan, M. de Ruiter, M. T. Alting, K. A. Macmillan, and M. F. Haase, Colloids and Surfaces A: Physicochemical and Engineering Aspects **666**, 131306 (2023).
